# Comorbidity Trajectories Associated With Alzheimer’s Disease: A Matched Case-Control Study in a United States Claims Database

**DOI:** 10.3389/fnins.2021.749305

**Published:** 2021-10-08

**Authors:** Lesley M. Butler, Richard Houghton, Anup Abraham, Maria Vassilaki, Gonzalo Durán-Pacheco

**Affiliations:** ^1^F. Hoffmann-La Roche Ltd., Basel, Switzerland; ^2^Genesis Research, Hoboken, NJ, United States; ^3^Department of Quantitative Health Sciences, Mayo Clinic, Rochester, MN, United States

**Keywords:** Alzheimer’s disease, comorbidity, principal component analysis, hierarchical cluster analysis, MarketScan, Medicare

## Abstract

**Background:** Trajectories of comorbidities among individuals at risk of Alzheimer’s disease (AD) may differ from those aging without AD clinical syndrome. Therefore, characterizing the comorbidity burden and pattern associated with AD risk may facilitate earlier detection, enable timely intervention, and help slow the rate of cognitive and functional decline in AD. This case-control study was performed to compare the prevalence of comorbidities between AD cases and controls during the 5 years prior to diagnosis (or index date for controls); and to identify comorbidities with a differential time-dependent prevalence trajectory during the 5 years prior to AD diagnosis.

**Methods:** Incident AD cases and individually matched controls were identified in a United States claims database between January 1, 2000 and December 31, 2016. AD status and comorbidities were defined based on the presence of diagnosis codes in administrative claims records. Generalized estimating equations were used to assess evidence of changes over time and between AD and controls. A principal component analysis and hierarchical clustering was performed to identify groups of AD-related comorbidities with respect to prevalence changes over time (or trajectory), and differences between AD and controls.

**Results:** Data from 186,064 individuals in the IBM MarketScan Commercial Claims and Medicare Supplementary databases were analyzed (93,032 AD cases and 93,032 non-AD controls). In total, there were 177 comorbidities with a ≥ 5% prevalence. Five main clusters of comorbidities were identified. Clusters differed between AD cases and controls in the overall magnitude of association with AD, in their diverging time trajectories, and in comorbidity prevalence. Three clusters contained comorbidities that notably increased in frequency over time in AD cases but not in controls during the 5-year period before AD diagnosis. Comorbidities in these clusters were related to the early signs and/or symptoms of AD, psychiatric and mood disorders, cerebrovascular disease, history of hazard and injuries, and metabolic, cardiovascular, and respiratory complaints.

**Conclusion:** We demonstrated a greater comorbidity burden among those who later developed AD vs. controls, and identified comorbidity clusters that could distinguish these two groups. Further investigation of comorbidity burden is warranted to facilitate early detection of individuals at risk of developing AD.

## Introduction

Alzheimer’s disease (AD) is the most common cause of dementia, accounting for 60–70% of cases. The prevalence of AD increases with age, with a global prevalence of 5–8% in people 60 years and older [World Health Organization [WHO], 2021]. While AD has previously been considered to have discrete and clearly defined clinical stages, it is now more usually considered to be a seamless continuum from an asymptomatic phase through a long preclinical period, to a symptomatic phase in which cognitive and then functional impairment become increasingly evident (Dubois et al., 2016; Aisen et al., 2017; Jack et al., 2018). Furthermore, while the terms “mild cognitive impairment (MCI)” or “prodromal AD (pAD)” and “mild AD” have traditionally been used in clinical trials to describe the early stages of AD, these are often studied together and referred to as “early AD” patients (Siemers, 2021).

Evidence suggests that treatment earlier in the disease continuum is likely to achieve greater disease modification and slow the rate of cognitive and functional decline (Dubois et al., 2016; Aisen et al., 2017; Jack et al., 2018). However, AD is only usually diagnosed once clinical symptoms become apparent, which may be as long as 15 years after the first pathological changes occur, leading to delays in treatment and potentially lost clinical benefit (Dubois et al., 2016; Aisen et al., 2017). Even after symptoms of AD become clinically evident, there exists a large population living with dementia who remain undiagnosed (Lang et al., 2017; Amjad et al., 2018; Genovese et al., 2018; Grandal Leiros et al., 2018). It is thought that among older adults with probable dementia (including AD), most (58.7%) were either undiagnosed (39.5%) or unaware of the diagnosis (19.2%) (Amjad et al., 2018). A meta-analysis of 23 studies conducted between 1988 and 2015 in community and residential settings reported a 61.7% pooled rate of undetected dementia (Lang et al., 2017).

This underdiagnosis may be due in part to a low dementia diagnosis rate in primary care (Boise et al., 1999; Geldmacher and Kerwin, 2013; Jørgensen et al., 2015; Lang et al., 2017; Small, 2017). Currently in the United States (US), a diagnosis of dementia in primary care is largely reliant on the self-presentation of a patient on the basis of symptoms or caregiver concerns (Iliffe et al., 1991; McCormick et al., 1994; Bradford et al., 2009), such that many cases go undiagnosed until late in the disease (McCormick et al., 1994). For those patients who do present, referral to a specialist then requires the primary care physician to act on a clinical suspicion (Brayne et al., 2007), which is itself prone to being missed or delayed (Iliffe et al., 1991; Callahan et al., 1995; Bradford et al., 2009). The availability of specialist tools to help evaluate whether a patient needs to be referred for specialist care may save time and expedite any decision-making process, potentially increasing the rate of diagnosis of AD.

Certain chronic medical conditions, including type 2 diabetes (T2DM), hypertension, coronary artery disease, and depression, are established risk factors for cognitive decline (Artero et al., 2008; Vicini Chilovi et al., 2009; Li et al., 2012; Roberts and Knopman, 2013; Imtiaz et al., 2014; Johnson et al., 2015; Vassilaki et al., 2015; Fan et al., 2017). These conditions are also common in multimorbidity (defined as at least two comorbid conditions) in older adults, which may also be associated with biomarkers of the preclinical AD stages (Sperling et al., 2011; Jack et al., 2014; Sperling et al., 2014) and suspected non-amyloid pathophysiology (Jack et al., 2016; Vassilaki et al., 2019), even before clinically detectable cognitive decline becomes apparent. Not only is there an increase in the prevalence of comorbidities among patients at risk of AD, but multimorbidity, a distinctive hallmark of aging and potentially a clinical marker of accelerated aging (Fabbri et al., 2015), is also associated with increased risk of cognitive impairment (Palmer et al., 2007; Vassilaki et al., 2015; Santiago and Potashkin, 2021). There is also evidence suggesting that the trajectories of comorbidities among individuals at risk of AD differ from those who are simply undergoing the normal process of aging (Oveisgharan and Hachinski, 2010; Velayudhan et al., 2010; Xu et al., 2010). Therefore, an evaluation of comorbidities and their trajectories during the early stage of disease is highly relevant in characterizing the natural history of AD dementia. In this way, identifying distinctive patterns of comorbidities, including signs and symptoms of early AD, may enable more timely cognitive assessment and specialist referral for an evaluation of AD diagnosis.

A data-driven approach was used in this analysis to identify comorbidities that occur before AD diagnosis that are associated with the development of AD. Incident AD cases and matched non-AD controls from the general population were identified in a US claims database and used to investigate comorbid diagnoses that occurred during the 5 years prior to a first diagnosis of AD. A window of 5 years to capture patients with early AD was set on the basis that the median duration between the onset of dementia-related symptoms and assessment or diagnosis is typically up to 3 years, according to literature reports (Boise et al., 1999; Knopman et al., 2000; Wackerbarth and Johnson, 2002; Wilkinson et al., 2004; Fiske et al., 2005; Speechly et al., 2008; Carpentier et al., 2010; van Vliet et al., 2013; Zhao et al., 2016). The methodology used in this analysis is a new application of a standard method used to identify patterns inherent in data.

This analysis has two primary objectives: (1) to compare the prevalence of comorbidities between AD cases and non-AD controls during the 5 years prior to diagnosis; and (2) to identify comorbidities with a time-dependent prevalence trajectory during the 5 years prior to AD diagnosis that is differential among cases, compared with controls.

## Materials and Methods

### Study Design and Setting

This was a retrospective, observational, case-control study conducted in the US using data from the IBM MarketScan^®^ Commercial Claims and Medicare Supplementary databases.

#### Study Population

**FIGURE 1:**
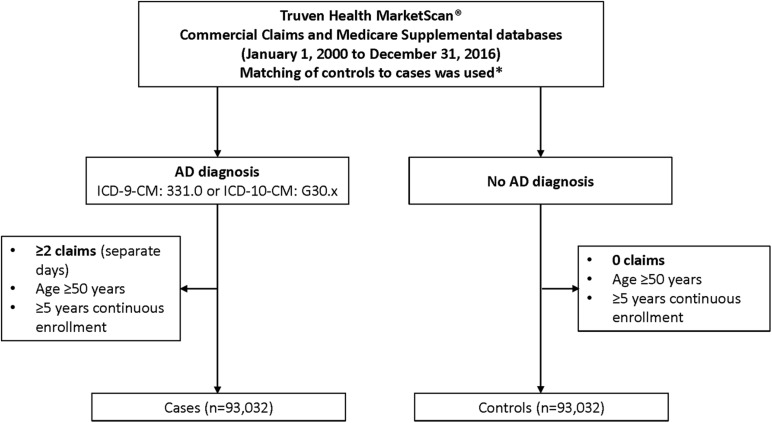
Flowchart of case inclusion process. *Cases were required to have at least two claims for AD on separate days at age ≥ 50 years (ICD-9-CM: 331.0 or ICD-10-CM: G30.x) and a 5-year period of continuous enrollment prior to first AD diagnosis, while eligible controls for each case were required to have no claims for AD (ICD-9-CM: 331.0 or ICD-10-CM: G30.x) during the 5-year window prior to the respective cases index date. Matching was based on sex; year of birth; insurance plan type at index; relationship to insurance plan holder; (previous) employment industry; and US region. AD, Alzheimer’s disease; ICD, International Classification of Diseases.

The study population consisted of individuals with AD (“cases”) and a matched group of individuals without AD (“controls”) (Figure 1). Cases were required to have at least two claims for AD on separate days at age ≥ 50 years [International Classification of Diseases (ICD)-9-CM: 331.0 or ICD-10-CM: G30.x] and a 5-year period of continuous enrollment prior to first AD diagnosis, while eligible controls for each case were required to have no claims for AD (ICD-9-CM: 331.0 or ICD-10-CM: G30.x) during the 5-year window prior to the respective cases index date. All eligible cases from the database were included in the analysis. The index date for cases was the first AD diagnosis date. For controls, the index date was set to the same date as the individually matched case.

#### Matching

For each AD case, a control (1:1) was selected randomly and without replacement from the pool of eligible controls, as defined above. Matching was based on sex; year of birth (hence also age, given the same index date); insurance plan type at index [e.g., Health Maintenance Organization (HMO); Preferred Provider Organization (PPO); Point of Service (POS); comprehensive]; relationship to insurance plan holder (employee or spouse/other); employment industry; and US region (West, Northeast, Midwest, or South).

#### Data Source

Data for this analysis were extracted from the IBM MarketScan^®^ Commercial Claims (“Commercial”) database and the Medicare Supplementary (“Medicare”) database. The commercial database contains active employees, early retirees, and dependents insured by employer−sponsored plans, while the Medicare database covers Medicare-eligible retirees (≥ 65 years) with employer-sponsored Medicare Supplementary plans. Both data sets were analyzed together in order to allow patients to be tracked from employment through into retirement.

Because the database is based on insurance claims, individuals are able to drop in and out of enrollment in the database. The “continuous enrollment period” was therefore defined on an individual-person level, based upon medical insurance coverage. Gaps in enrollment of up to 62 days (2 months) were allowed so long as this gap was contained by periods of documented enrollment before and afterward. Continuous enrollment periods had maximum boundaries of the study period (January 1, 2000 to December 31, 2016).

#### Comorbidity Definitions

The presence of individual comorbidities was evaluated in each of the 5-yearly intervals prior to AD diagnosis, based upon the occurrence of at least one diagnosis claim (code) in the relevant time period. Diagnosis recorded in the database was based upon the International Classification of Diseases, Ninth Revision, Clinical Modification (ICD-9-CM) up until 30 September 2015, and thereafter was based on ICD-10-CM criteria. For the purposes of this study, all ICD-10-CM codes were converted to ICD-9-CM prior to further grouping.

Comorbidities were grouped into approximately 1,200 categories, based upon the first three digits of each ICD-9-CM code. The decodes for these three-digit sub-chapters can be found in the “icd” r package (Wasey et al., 2021). Comorbidities with a total 5-year occurrence of ≥5% (across both AD cases and controls, combined) were kept.

#### Ethical Considerations

This was a retrospective, observational study of secondary use data. All personal information used was de-identified (with no possibility of linkage back to individual identified patients) and is compliant with the US Health Insurance Portability and Accountability Act (HIPAA).

### Statistical Analysis

#### Descriptive Analyses

Demographic/personal characteristics of AD cases and non-AD controls from both cohorts were summarized using means and standard deviations (SDs) for continuous variables and frequencies and percentages for categorical variables. For both AD cases and non-AD controls, the proportion of patients with each comorbidity with ≥ 5% prevalence, over the 5 years prior to index date, were reported.

#### Associations Between Alzheimer’s Disease and Comorbidities

Generalized estimating equations (GEE) were used to estimate the odds of each comorbidity as a function of time (yearly intervals prior to index), AD diagnosis status, and their interaction. Odds ratios (ORs) were estimated, and hypothesis tests were conducted on the following model:

logit(p)ijk=η+iαADi+jβTimei+kγADiTimejk

where pijk = proportion of subjects who reported a claim for comorbidity i at year k prior index date (*k* = 1,…, 5), having AD diagnosis status (*j* = 1, 2). η is the logit’s general mean, α is the log odds of AD vs. control, β is the change in the log odds by change in 1 year, and γ is the difference of log odds changes per year between AD cases and controls. Standard errors were computed using the sandwich robust variance estimator (Liang and Zeger, 1986), assuming an unstructured within-subject covariance matrix. Multiple testing correction (False Discovery Rate) was applied to account for multiple testing across comorbidities.

#### Multivariate Analysis: Principal Component Analysis and Hierarchical Cluster Analysis

In order to identify groups of comorbidities associated with AD that varied in terms of frequency changes over time and between AD and non-AD control groups, four metrics from the GEE models were considered; two denoting the magnitude of the differences between AD vs. controls (#1 and #2) and two related to the level of evidence of such differences (#3 and #4): (1) the difference in log odds of AD vs. non-AD controls (i.e., coefficient α centered at the mean follow-up time prior to AD diagnosis); (2) the log_10_ scaled *p*-value associated with the hypothesis test of the centered α; (3) the interaction term, γ, which denotes the difference of slopes between changes over time of AD patients vs. controls; (4) the log_10_ scaled *p*-value associated with the hypothesis test of γ. Metrics 1 and 2 assessed the overall difference in AD vs. controls comorbidities over the period prior to index date, whereas metrics 3 and 4 assessed the difference in the comorbidity trajectory over time between AD and controls, during the 5-year period prior to index date.

A data matrix of dimensions n (number of comorbidities) and *p* = 4 (the four metrics selected) was created and submitted to PCA, where a new set of orthogonal variables were obtained. The new data matrix was analyzed by hierarchical clustering with Ward’s grouping algorithm along with Euclidean distances in order to identify AD comorbidities by their changes over time and between groups.

## Results

### Demographic Characteristics

Data from 186,064 individuals in the IBM MarketScan^®^ Commercial Claims and Medicare Supplementary databases were analyzed (93,032 AD cases and 93,032 non-AD controls) (Table 1). Overall, 59% of the population was female. The study population was predominantly older adults, with an average age of 82 years. Seventeen percent of participants were aged 90 years or older. The majority of the included population (57%) had comprehensive insurance, followed by PPO (32%). Most participants resided in the North-Central US region (45%), followed by the Southern states (29%).

**TABLE 1:** Demographic characteristics.

Variable	Overall *N* = 186,064	Cases *N* = 93,032	Controls *N* = 93,032
**Sex, % female**	59.12	59.12	59.12
**Age, years, mean (*SD*)**	82.11 (8.1)	82.11 (8.1)	82.11 (8.1)
**Age, years**			
50–59, %	1.3	1.3	1.3
60–69, %	5.9	5.9	5.9
70–79, %	25.7	25.7	25.7
80–89, %	50.3	50.3	50.3
90+, %	16.8	16.8	16.8
**Region, %**			
North-Central	45.4	45.4	45.4
South	29.2	29.2	29.2
West	14.4	14.4	14.4
Northeast	10.8	10.8	10.8
Unknown	0.2	0.2	0.2
**Plan holder %**			
Current/previous employee	81.8	81.8	81.8
Spouse/child/other	18.2	18.2	18.2
**Healthcare plan type %**			
Comprehensive	56.9	56.9	56.9
PPO	32.5	32.5	32.5
HMO	6.0	6.0	6.0
POS	3.2	3.2	3.2
Other	0.8	0.8	0.8
Missing	0.8	0.8	0.8
**Industry, n (%)**			
Manufacturing, durable goods	42.7	42.7	42.7
Transportation, communications, utilities	18.1	18.1	18.1
Services	11.1	11.1	11.1
Other and missing	28.1	28.1	28.1

*CDHP, Consumer Driven Health Plan; EPO, Exclusive Provider Organization; HDHP, High Deductible Health Plan; HMO, Health Maintenance Organization; POS, Point of Service; PPO, Preferred Provider Organization; SD, standard deviation.*

### Prevalence and Association of Comorbidities, Signs, and Symptoms During 5 Years Prior to Alzheimer’s Disease Diagnosis

In total, 177 comorbidities were identified with a prevalence of ≥5% (Supplementary Table 1). Of these, the individual comorbidities [(ICD-9 code; prevalence (%)] with the highest 5-year pooled prevalence prior to index date across AD cases and in controls were: essential hypertension (401; 74.5%), general symptoms (780; 66.9%), symptoms involving the respiratory system (786; 65.6%), disorders of lipoid metabolism (272; 54.8%), and other and unspecified disorders of joint (719; 53.3%) (Table 2). However, the comorbidities with highest ORs in AD cases compared with controls in the period prior to index date were: persistent mental disorders due to conditions classified elsewhere (294), other non-organic psychoses (298), other cerebral degenerations (331), transient mental disorders due to conditions classified elsewhere (293), general symptoms (780), other conditions of the brain (348), episodic mood disorders (296), and depressive disorders not elsewhere classified (331), among others (Supplementary Figure 1).

**TABLE 2:** Comorbidities with the highest pooled prevalence across cases and control.

Description	Overall*N* = 186,064%	Cases*N* = 93,032%	Controls*N* = 93,032%
Essential hypertension	74.5	83.6	65.4
General symptoms*	66.9	86.3	47.5
Symptoms involving respiratory system and other chest symptoms	65.6	75.8	55.5
Disorders of lipoid metabolism	54.8	62.2	47.3
Other and unspecified disorders of joint	53.3	63.5	43.2
Other disorders of soft tissues	44.8	53.6	36.1
Cataract	44.1	48.8	39.4
Osteoarthrosis and allied disorders	43.0	50.5	35.5
Other and unspecified disorders of back	38.7	45.7	31.7
Cardiac dysrhythmias	38.5	44.8	32.2
Other symptoms involving abdomen and pelvis	37.8	45.0	30.6
Special screening for malignant neoplasms	37.2	40.5	33.9
Other disorders of urethra and urinary tract	36.4	45.4	27.4
Other forms of chronic ischemic heart disease	35.5	40.7	30.3
Symptoms involving digestive system	34.5	42.6	26.5
Encounter for other and unspecified procedures and aftercare	34.3	40.0	28.5
Other disorders of bone and cartilage	32.4	37.7	27.0
Other dermatoses	31.9	33.7	30.1
Symptoms involving skin and other integumentary tissue	31.7	38.3	25.1
Special investigations and examinations	31.3	34.9	27.8

*Comorbidities are arranged in descending order of prevalence in the overall population.*

*The frequency (%) of general symptoms were: Alteration of consciousness (22.5), hallucinations (2.1), syncope and collapse (30.3), convulsions (7.6), dizziness and giddiness (38.0), sleep disturbances (16.8), Fever and other physiologic disturbances of temperature regulation (14.9), malaise and fatigue (54.3), generalized hyperhidrosis (1.7), and other general symptoms (52.0).

*CI, confidence interval.*

### Multivariate Analysis

**FIGURE 2:**
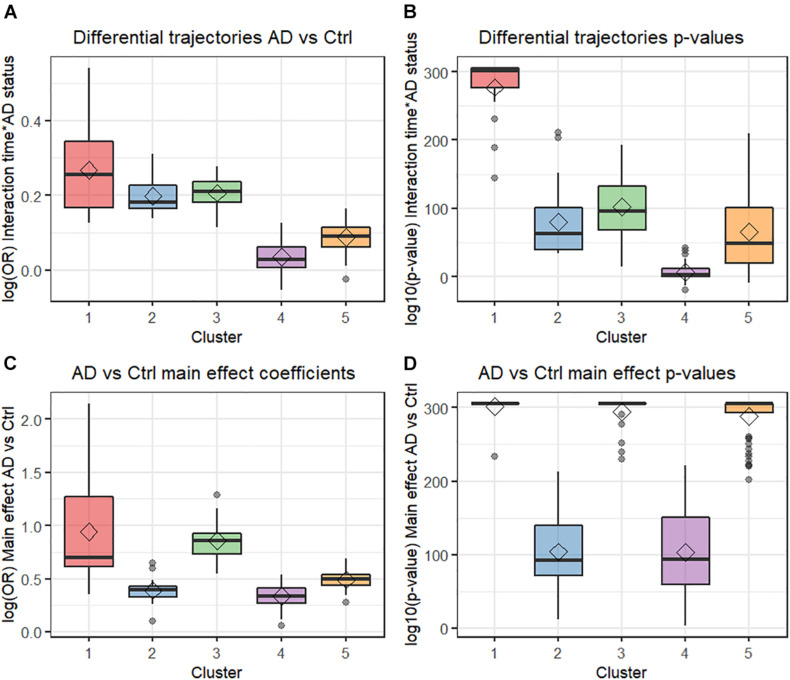
Box plots from multivariate analysis. **(A)** Differential trajectories AD vs. control. **(B)** Differential trajectories *p*-values. **(C)** AD vs. control effect coefficients. **(D)** AD vs. control main effect *p*-values. AD, Alzheimer’s disease; Ctr, Control; OR, Odds ratio.

Four principal components (PCs) were obtained from the PCA analysis conducted on the four metrics used to differentiate comorbidities. The first, second, third, and fourth PCs explained 70.3, 15.4, 11.0, and 3.3% of variance in the data, respectively. Supplementary Figures 2A,B display the distribution of the five clusters of comorbidities in biplots for the first and second PCs and in the first and third PCs, respectively. Five main clusters of comorbidities were found from the hierarchical cluster analysis conducted on the four PCs (Figure 2 and Supplementary Figure 3). Clusters 1, 3, and 5 consisted of comorbidities with higher ORs and smaller *p*-values for the comparison of AD vs. controls mid-term prior to index date (Figures 2C,D). Although clusters 1, 2, and 3 contained comorbidities with higher ORs for the differential time trajectories between AD vs. controls, cluster 1 stood out as the collection of comorbidities with the largest differential time-dependent trajectories among AD vs. controls and smaller *p*-values for the interaction terms (Figures 2A,B).

### Description of the Clusters, Differential Trajectories of Comorbidities, Signs, and Symptoms During 5 Years Prior to Alzheimer’s Disease Diagnosis Among Cases vs. Controls

The comorbidities included under each cluster are listed in Supplementary Table 1, trajectories are shown in Supplementary Figure 4.

Cluster 1 contained 18 comorbidities with a large difference in trajectories, increasing rapidly in the AD group prior to diagnosis, but not in the controls (Supplementary Figure 4A). Comorbidities were quite prevalent overall (generally >15% in AD and non-AD combined groups). This cluster included terms such as persistent mental disorder (294), non-organic psychoses (298), transient mental disorders (293), and other cerebral degenerations (331), but also included other wide-ranging comorbidities such as fluid and electrolyte imbalance (276), symptoms of respiratory system (786), heart failure (428), and dermatophystosis (110) (Supplementary Figure 4A and Supplementary Table 1).

**FIGURE 3:**
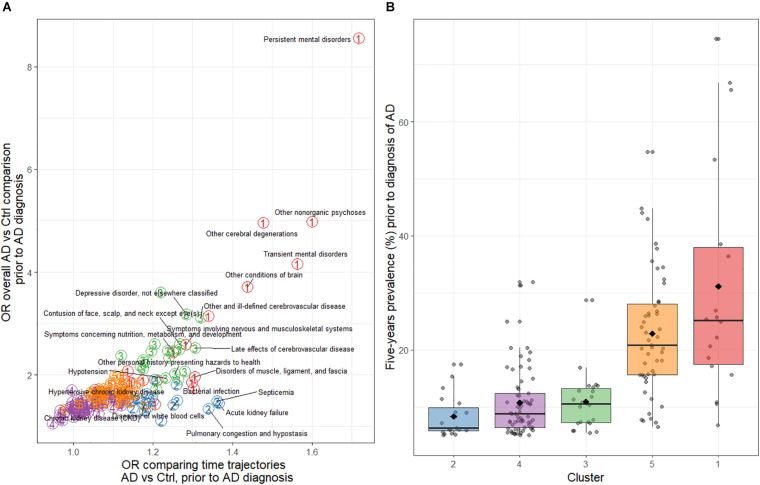
**(A)** Odds ratios for the overall AD cases vs. non-AD controls comparison of comorbidities and for the time trajectories of comorbidities during the 5-year period before AD diagnosis. The colored circled numbers refer to the cluster that the comorbidity belongs to (i.e., red-colored 1 is cluster 1, etc.). **(B)** Distribution of the average comorbidity prevalence during the 5-year period before AD diagnosis by cluster. AD, Alzheimer’s disease; Ctr, Control.

Cluster 2 contained 19 comorbidities with marked differences in time trajectories between AD vs. non-AD (Supplementary Figure 4B), lower OR for the overall AD vs. non-AD comparison in the period prior to AD diagnosis (Figure 3A), and with lower overall prevalence (generally <15% in AD and non-AD combined, Figure 3B). This cluster included diseases of the kidneys, such as chronic (585) and acute (584), and other (593) kidney disease; and lungs, such as emphysema (492), pneumonia (486), and chronic bronchitis (491). Other comorbidities not related to lung or kidney were also included, such as bacterial infections (Wilkinson et al., 2004), septicemia (Speechly et al., 2008), vertebral fractures (805), and diseases of white blood cells (288) (Supplementary Figure 4B and Supplementary Table 1).

Cluster 3 contained 22 comorbidities, also with large trajectory differences between AD cases and controls (Supplementary Figure 4C). The difference with cluster 1 was that the statistical significance of the trajectory differences were not as strong and that comorbidities in this group had lower prevalence (Figure 3B). Comorbidities included psychiatric comorbidities such as depression (311) and anxiety (300) but also included terms related to care and rehabilitation procedures (V57), personal history of hazard to health (V15), nutrition, metabolism and development (783), fracture of femur (820), open wound of head (873), and contusions (of various body parts; 920 and 922–924). Additionally, various cerebrovascular disease comorbidities were included (434–438) (Supplementary Table 1).

Cluster 4 was the largest group with 65 comorbidities. This cluster included comorbidities with the most similar trajectories over time (Figure 3A and Supplementary Figure 4D), and a more similar prevalence between cases and controls. In addition, some comorbidities became less prevalent in both groups over time (e.g., benign neoplasm of skin; 216).

Cluster 5 comorbidities had both trajectory differences and overall AD vs. non-AD prevalence differences that were more subtle than shown for comorbidities in cluster 3 (Figure 3A and Supplementary Figure 4E), despite the relatively high prevalence of some symptoms such as symptoms of the digestive system (787), osteoarthritis (715), disorders of lipoid metabolism (272), and cataract (366). This cluster was also large (51 comorbidities), and contained a more heterogeneous group of comorbidities, both in terms of prevalence and the affected system and/or organ (Figure 3B and Supplementary Table 1).

## Discussion

This project used a novel application of longitudinal data modeling and multivariate analysis to identify clusters of comorbidities that occur in patients’ records prior to diagnosis with AD. We identified clusters of AD comorbidities that may diverge in terms of frequency changes over time and between AD and non-AD control groups.

Cluster 1 offered the largest overall differences between AD cases and controls prior to diagnosis and, moreover, differential trajectories over time in AD cases compared with controls. The comorbidities in cluster 1 were fairly prevalent overall (>15% in AD and non-AD combined). Some comorbidities could be related to the early signs/symptoms of AD, such as persistent mental disorders and other cerebral degenerations (including MCI). However, also in cluster 1, with similar prevalence and trajectory differences prior to AD diagnosis, were serious comorbidities of other organs classes such as the respiratory and cardiovascular systems. These comorbidity classes have previously been shown to be associated with progression (Jutkowitz et al., 2017a,b; Koskas et al., 2017) and risk of AD and dementia (Bauer et al., 2014; Ruthirakuhan et al., 2019).

Similarly, other comorbidities in clusters 2, 3, and 5 showed differences between cases and controls, even if not directly related to AD. This shows that the comorbidity burden starts years prior to AD, during the early AD or MCI phase of progression or even earlier. For example, not only were relatively less frequent comorbidities of the kidneys and lungs (among others) more prevalent prior to AD diagnosis (cluster 2), but so were depression, anxiety, and comorbidities related to accidents or injuries (for example, history of personal hazards, open head wounds, and contusion to various body parts; cluster 3). These comorbidities have been associated with lower health-related quality of life in patients with AD (Barbe et al., 2018), as well as risk factors for cognitive impairment (Krell-Roesch et al., 2021). Falls are considered a marker of cognitive impairment, and an increased risk of falls has been reported among adults within the early, preclinical stage of AD (Stark et al., 2013). Together these and our findings reflect the need for additional measures to ensure patient safety around the home or in care facilities, respectively.

A potential limitation of this study is that comorbidities may be recorded more frequently in some patients’ records, simply due to more encounters with the healthcare system during the work up of an AD diagnosis. However, cluster 4 was the largest group (65 comorbidities), and contains comorbidities without large differences between cases and controls. Small imbalances that do remain in cluster 4 are much lower in magnitude than for the differences between cases and controls in the other clusters, thus providing evidence of true elevated comorbidity burden in early AD, above any systematic differences due to reporting bias.

There is a real need to better characterize patients either at risk of developing AD or with AD early in the course of their disease, to allow early intervention that could slow the rate of cognitive and functional decline (Dubois et al., 2016; Aisen et al., 2017; Jack et al., 2018). However, AD still tends to be diagnosed at a relatively advanced stage, meaning that the opportunity for early intervention is lost (Bature et al., 2018; Barnes et al., 2020).

AD usually has a slow progression (No authors listed, 2020), and although what defines AD as a unique neurodegenerative disease (among others conditions that could lead to dementia) are the β-amyloid plaques and the neurofibrillary tau deposits (Jack et al., 2018), there is still a lot of work to be done to delineate the AD pathogenesis causal pathways. Although, not all current findings are amenable to an easy interpretation, novel research (Wang et al., 2021) suggests that multiple pathological pathways could be involved in AD pathogenesis such as unresolved neuroinflammation, abnormal glucose metabolism, vascular alterations, mitochondria dysfunction; pathological processes present in many comorbidities (e.g., vascular conditions, diabetes, infections) in the present study.

Considerable research has been devoted over recent years to the use of biomarkers to diagnose AD early, and this approach has shown promising results (Frisoni et al., 2017; Blennow and Zetterberg, 2018; Giorgio et al., 2020). However, as most cases of AD are diagnosed in primary care, it is important to have a simple and convenient tool that is readily available to GPs to identify patients who may be at risk of progressing to AD dementia (Iliffe et al., 1991; McCormick et al., 1994; Bradford et al., 2009). It may be possible in a primary care setting to flag a patient’s chart if a pattern of comorbidities is observed within a short period of time. This would then prompt healthcare professionals to inquire about memory concerns and possibly refer the patient to a specialist for cognitive testing and/or any imaging or fluid biomarkers available, including, but not limited to, magnetic resonance imaging (MRI), positron emission tomography (PET), and/or cerebrospinal fluid (CSF) Aβ and tau tests (Leocadi et al., 2020; Leuzy et al., 2021). The results of the current study add to and expand previous work toward the development of such a tool.

The study findings need to be viewed in light of the following limitations. There was an opportunity for misclassification of AD status among cases in this study because status was defined only by the presence of a diagnosis code for AD and not any biomarker or pathology data. Thus, the AD cases in this study are likely to have “Alzheimer’s clinical syndrome,” or what has been previously referred to as “clinically probably AD” (Jack et al., 2018). Another potential reason for misclassification of AD status is that the study cohort is skewed toward older age where seventeen percent are 90 years or older. It is possible that the older AD cases actually have other forms of dementia and/or neurodegenerative disorders with similar clinical presentation to AD and that are more common in older adults, such as limbic-predominant age-related TDP-43 encephalopathy (Nelson et al., 2019). Other forms of neurodegenerative disorders may be associated with a unique set of comorbidities, confounding interpretations. The statistical analysis also has a number of limitations, including the hierarchical clustering used to define groups. Although we used robust distance metrics on orthogonal data coordinates, alternative grouping-algorithms might have achieved different groupings. In addition, patients were grouped at the 3-digit ICD level as a short-hand for the more detailed patient histories collected. Although further refinement to lower-level codes may have yielded insights on more specific comorbidities, the scope of this study using an exploratory statistical approach was best suited for analyses with the higher-level groupings. Another potential limitation is that clusters were defined based on metrics related to overall differences in prevalence and trajectories; although our model assumed linear trajectories over time, and *p*-values are dependent on prevalence of comorbidities, not only the effect size. Finally, all comorbidities with prevalence < 5% across cases and controls were excluded, and no terms that captured associations other than linear shapes (or differences) were included based on the four metrics described in this study.

Strengths of the study include the large study size and follow up, taken from objectively and systematically collected data sources. Cases and controls were matched based on a number of factors including sex; year of birth; insurance plan type at index date; relationship to insurance plan holder; (previous) employment industry; and US region.

## Conclusion

Although we demonstrated a greater comorbidity burden among those who later developed AD (vs. those who did not), it cannot be ruled out that the observed relationship between comorbidity burden and AD was due in part to residual confounding by underlying factors and/or mechanisms related to aging, given that multimorbidity is associated with accelerated aging (Fabbri et al., 2015). We also identified clusters of comorbidities that could distinguish AD cases and non-cases. Further investigation of comorbidity clusters is warranted to facilitate early detection of individuals at risk of developing AD.

## Data Availability Statement

The data analyzed in this study is subject to the following licenses/restrictions: Commercial claims databases are available with subscription payment. Requests to access these datasets should be directed to RH, richard.houghton@roche.com.

## Author Contributions

LB, RH, and GD-P developed the study concept, protocol, wrote the manuscript, and statistical analysis plan. LB, RH, AA, GD-P, and MV assisted in the interpretation of the findings and provided critical revision to the manuscript. GD-P and AA performed the statistical analyses. All authors read and approved the final manuscript.

## Conflict of Interest

LB, RH, and GD-P were full-time employees of F. Hoffmann-La Roche Ltd., AA was a full-time employee of Genesis Research and received funding from F Hoffmann-La Roche Ltd., for work on this study. MV currently consults for Roche, receives research funding from NIH/NIA, and has equity ownership in Abbott Laboratories, Johnson and Johnson, Medtronic, and Amgen. The funders were not involved in the study design, collection, analysis, interpretation of data, the writing of this article or the decision to submit it for publication.

## Publisher’s Note

All claims expressed in this article are solely those of the authors and do not necessarily represent those of their affiliated organizations, or those of the publisher, the editors and the reviewers. Any product that may be evaluated in this article, or claim that may be made by its manufacturer, is not guaranteed or endorsed by the publisher.

## References

[B1] Aisen P. S. Cummings J. Jack C. R. Jr. Morris J. C. Sperling R. Frölich L. (2017). On the path to 2025: understanding the Alzheimer’s disease continuum. *Alzheimers Res. Ther.* 9:60. 28793924 10.1186/s13195-017-0283-5 PMC5549378

[B2] Amjad H. Roth D. L. Sheehan O. C. Lyketsos C. G. Wolff J. L. Samus Q. M. (2018). Underdiagnosis of dementia: an observational study of patterns in diagnosis and awareness in US older adults. *J. Gen. Intern. Med.* 33 1131–1138. 10.1007/s11606-018-4377-y 29508259 PMC6025653

[B3] Artero S. Ancelin M. L. Portet F. Dupuy A. Berr C. Dartigues J. F. (2008). Risk profiles for mild cognitive impairment and progression to dementia are gender specific. *J. Neurol. Neurosurg. Psychiatry* 79 979–984. 10.1136/jnnp.2007.136903 18450788

[B4] Barbe C. Jolly D. Morrone I. Wolak-Thierry A. Dramé M. Novella J. L. (2018). Factors associated with quality of life in patients with Alzheimer’s disease. *BMC Geriatr.* 18:159. 10.1186/s12877-018-0855-7 29986669 PMC6038200

[B5] Barnes D. E. Zhou J. Walker R. L. Larson E. B. Lee S. J. Boscardin W. J. (2020). Development and validation of eRADAR: a tool using EHR data to detect unrecognized dementia. *J. Am. Geriatr. Soc.* 68 103–111. 10.1111/jgs.16182 31612463 PMC7094818

[B6] Bature F. Guinn B. Pang D. Pappas Y. (2018). Perspectives of general practitioners on the issues surrounding the late diagnosis of Alzheimer’s disease. *J. Alzheimers Dis. Rep.* 2 207–212. 10.3233/adr-180064 30560245 PMC6294576

[B7] Bauer K. Schwarzkopf L. Graessel E. Holle R. (2014). A claims data-based comparison of comorbidity in individuals with and without dementia. *BMC Geriatr.* 14:10. 10.1186/1471-2318-14-10 24472217 PMC3909381

[B8] Blennow K. Zetterberg H. (2018). Biomarkers for Alzheimer’s disease: current status and prospects for the future. *J. Intern. Med.* 284 643–663. 10.1111/joim.12816 30051512

[B9] Boise L. Camicioli R. Morgan D. L. Rose J. H. Congleton L. (1999). Diagnosing dementia: perspectives of primary care physicians. *Gerontologist* 39 457–464. 10.1093/geront/39.4.457 10495584

[B10] Bradford A. Kunik M. E. Schulz P. Williams S. P. Singh H. (2009). Missed and delayed diagnosis of dementia in primary care: prevalence and contributing factors. *Alzheimer Dis. Assoc. Disord.* 23 306–314. 10.1097/wad.0b013e3181a6bebc 19568149 PMC2787842

[B11] Brayne C. Fox C. Boustani M. (2007). Dementia screening in primary care: is it time? *JAMA* 298 2409–2411. 10.1001/jama.298.20.2409 18042918

[B12] Callahan C. M. Hendrie H. C. Tierney W. M. (1995). Documentation and evaluation of cognitive impairment in elderly primary care patients. *Ann. Intern. Med.* 122 422–429. 10.7326/0003-4819-122-6-199503150-00004 7856990

[B13] Carpentier N. Bernard P. Grenier A. Guberman N. (2010). Using the life course perspective to study the entry into the illness trajectory: the perspective of caregivers of people with Alzheimer’s disease. *Soc. Sci. Med.* 70 1501–1508. 10.1016/j.socscimed.2009.12.038 20207459 PMC5123874

[B14] Dubois B. Hampel H. Feldman H. H. Scheltens P. Aisen P. Andrieu S. (2016). Preclinical Alzheimer’s disease: definition, natural history, and diagnostic criteria. *Alzheimers Dement* 12 292–323. 27012484 10.1016/j.jalz.2016.02.002 PMC6417794

[B15] Fabbri E. Zoli M. Gonzalez-Freire M. Salive M. E. Studenski S. A. Ferrucci L. (2015). Aging and multimorbidity: new tasks, priorities, and frontiers for integrated gerontological and clinical research. *J. Am. Med. Dir. Assoc.* 16 640–647. 10.1016/j.jamda.2015.03.013 25958334 PMC5125299

[B16] Fan Y. C. Hsu J. L. Tung H. Y. Chou C. C. Bai C. H. (2017). Increased dementia risk predominantly in diabetes mellitus rather than in hypertension or hyperlipidemia: a population-based cohort study. *Alzheimers Res. Ther.* 9:7. 10.1186/s13195-017-0236-z 28162091 PMC5292809

[B17] Fiske A. Gatz M. Aadnøy B. Pedersen N. L. (2005). Assessing age of dementia onset: validity of informant reports. *Alzheimer Dis. Assoc. Disord.* 19 128–134. 10.1097/01.wad.0000174947.76968.74 16118529

[B18] Frisoni G. B. Boccardi M. Barkhof F. Blennow K. Cappa S. Chiotis K. (2017). Strategic roadmap for an early diagnosis of Alzheimer’s disease based on biomarkers. *Lancet Neurol.* 16 661–676. 10.1016/S1474-4422(17)30159-X 28721928

[B19] Geldmacher D. S. Kerwin D. R. (2013). Practical diagnosis and management of dementia due to Alzheimer’s disease in the primary care setting: an evidence-based approach. *Prim Care Companion CNS Disord* 15:PCC.12r01474. 10.4088/PCC.12r01474 PMC3869604 24392252

[B20] Genovese M. C. Fleischmann R. Combe B. Hall S. Rubbert-Roth A. Zhang Y. (2018). Safety and efficacy of upadacitinib in patients with active rheumatoid arthritis refractory to biologic disease-modifying anti-rheumatic drugs (SELECT-BEYOND): a double-blind, randomised controlled phase 3 trial. *Lancet* 391 2513–2524. 10.1016/s0140-6736(18)31116-4 29908670

[B21] Giorgio J. Landau S. M. Jagust W. J. Tino P. Kourtzi Z. (2020). Modelling prognostic trajectories of cognitive decline due to Alzheimer’s disease. *Neuroimage Clin.* 26:102199. 10.1016/j.nicl.2020.102199 32106025 PMC7044529

[B22] Grandal Leiros B. Pérez Méndez L. I. Zelaya Huerta M. V. Moreno Eguinoa L. García-Bragado F. Tuñón (2018). Prevalence and concordance between the clinical and the post-mortem diagnosis of dementia in a psychogeriatric clinic. *Neurología (English Edition)* 33 13–17. 10.1016/j.nrleng.2016.04.004 27328891

[B23] Iliffe S. Haines A. Gallivan S. Booroff A. Goldenberg E. Morgan P. (1991). Assessment of elderly people in general practice. 1. social circumstances and mental state. *Br. J. Gen. Pract.* 41 9–12. 1805805 PMC1371476

[B24] Imtiaz B. Tolppanen A. M. Kivipelto M. Soininen H. (2014). Future directions in Alzheimer’s disease from risk factors to prevention. *Biochem. Pharmacol.* 88 661–670. 10.1016/j.bcp.2014.01.003 24418410

[B25] Jack C. R. Jr. Bennett D. A. Blennow K. Carrillo M. C. Dunn B. Haeberlein S. B. (2018). NIA-AA research framework: toward a biological definition of Alzheimer’s disease. *Alzheimers Dement* 14 535–562. 29653606 10.1016/j.jalz.2018.02.018 PMC5958625

[B26] Jack C. R. Jr. Knopman D. S. Chételat G. Dickson D. Fagan A. M. Frisoni G. B. (2016). Suspected non-Alzheimer disease pathophysiology–concept and controversy. *Nat. Rev. Neurol.* 12 117–124. 10.1038/nrneurol.2015.251 26782335 PMC4784257

[B27] Jack C. R. Jr. Wiste H. J. Weigand S. D. Rocca W. A. Knopman D. S. Mielke M. M. (2014). Age-specific population frequencies of cerebral β-amyloidosis and neurodegeneration among people with normal cognitive function aged 50-89 years: a cross-sectional study. *Lancet Neurol.* 13 997–1005. 10.1016/s1474-4422(14)70194-2 25201514 PMC4324499

[B28] Johnson L. A. Gamboa A. Vintimilla R. Cheatwood A. J. Grant A. Trivedi A. (2015). Comorbid depression and diabetes as a risk for mild cognitive impairment and Alzheimer’s disease in elderly mexican americans. *J. Alzheimers. Dis.* 47 129–136. 10.3233/jad-142907 26402761 PMC13270981

[B29] Jørgensen T. S. Torp-Pedersen C. Gislason G. H. Andersson C. Holm E. (2015). Time trend in Alzheimer diagnoses and the association between distance to an Alzheimer clinic and Alzheimer diagnosis. *Eur. J. Public Health* 25 522–527. 10.1093/eurpub/cku118 25085468

[B30] Jutkowitz E. Kane R. L. Dowd B. Gaugler J. E. MacLehose R. F. Kuntz K. M. (2017a). Effects of cognition, function, and behavioral and psychological symptoms on medicare expenditures and health care utilization for persons with dementia. *J. Gerontol. A Biol. Sci. Med. Sci.* 72 818–824. 28369209 10.1093/gerona/glx035 PMC6075208

[B31] Jutkowitz E. Kuntz K. M. Dowd B. Gaugler J. E. MacLehose R. F. Kane R. L. (2017b). Effects of cognition, function, and behavioral and psychological symptoms on out-of-pocket medical and nursing home expenditures and time spent caregiving for persons with dementia. *Alzheimers Dement* 13 801–809. 28161279 10.1016/j.jalz.2016.12.011 PMC5644025

[B32] Knopman D. Donohue J. A. Gutterman E. M. (2000). Patterns of care in the early stages of Alzheimer’s disease: impediments to timely diagnosis. *J. Am. Geriatr. Soc.* 48 300–304. 10.1111/j.1532-5415.2000.tb02650.x 10733057

[B33] Koskas P. Henry-Feugeas M. C. Feugeas J. P. Ou P. Drunat O. (2017). Factors of rapid cognitive decline in late onset Alzheimer’s disease. *Curr. Aging Sci.* 10 129–135. 10.2174/1874609810666170102143257 28042772

[B34] Krell-Roesch J. Syrjanen J. A. Machulda M. M. Christianson T. J. Kremers W. K. Mielke M. M. (2021). Neuropsychiatric symptoms and the outcome of cognitive trajectories in older adults free of dementia: the mayo clinic study of aging. *Int. J. Geriatr. Psychiatry* 36 1362–1369. 33724517 10.1002/gps.5528 PMC8451750

[B35] Lang L. Clifford A. Wei L. Zhang D. Leung D. Augustine G. (2017). Prevalence and determinants of undetected dementia in the community: a systematic literature review and a meta-analysis. *BMJ Open* 7:e011146. 10.1136/bmjopen-2016-011146 28159845 PMC5293981

[B36] Leocadi M. Canu E. Calderaro D. Corbetta D. Filippi M. Agosta F. (2020). An update on magnetic resonance imaging markers in AD. *Ther. Adv. Neurol. Disord.* 13:1756286420947986. 33747128 10.1177/1756286420947986 PMC7903819

[B37] Leuzy A. Ashton N. J. Mattsson-Carlgren N. Dodich A. Boccardi M. Corre J. (2021). 2020 update on the clinical validity of cerebrospinal fluid amyloid, tau, and phospho-tau as biomarkers for Alzheimer’s disease in the context of a structured 5-phase development framework. *Eur. J. Nucl. Med. Mol. Imaging* 48 2121–2139. 10.1007/s00259-021-05258-7 33674895 PMC8175301

[B38] Li L. Wang Y. Yan J. Chen Y. Zhou R. Yi X. (2012). Clinical predictors of cognitive decline in patients with mild cognitive impairment: the Chongqing aging study. *J. Neurol.* 259 1303–1311. 10.1007/s00415-011-6342-0 22186849

[B39] Liang K.-Y. Zeger S. L. (1986). Longitudinal data analysis using generalized linear models. *Biometrika* 73 13–22. 10.1093/biomet/73.1.13

[B40] McCormick W. C. Kukull W. A. van Belle G. Bowen J. D. Teri L. Larson E. B. (1994). Symptom patterns and comorbidity in the early stages of Alzheimer’s disease. *J. Am. Geriatr. Soc.* 42 517–521. 10.1111/j.1532-5415.1994.tb04974.x 8176147

[B41] Nelson P. T. Dickson D. W. Trojanowski J. Q. Jack C. R. Boyle P. A. Arfanakis K. (2019). Limbic-predominant age-related TDP-43 encephalopathy (LATE): consensus working group report. *Brain* 142 1503–1527. 31039256 10.1093/brain/awz099 PMC6536849

[B42] No authors listed (2020). 2020 Alzheimer’s disease facts and figures. *Alzheimers Dement.* Online ahead of print. 10.1002/alz.12068 32157811

[B43] Oveisgharan S. Hachinski V. (2010). Hypertension, executive dysfunction, and progression to dementia: the canadian study of health and aging. *Arch. Neurol.* 67 187–192. 20142526 10.1001/archneurol.2009.312

[B44] Palmer K. Berger A. K. Monastero R. Winblad B. Bäckman L. Fratiglioni L. (2007). Predictors of progression from mild cognitive impairment to Alzheimer disease. *Neurology* 68 1596–1602. 10.1212/01.wnl.0000260968.92345.3f 17485646

[B45] Roberts R. Knopman D. S. (2013). Classification and epidemiology of MCI. *Clin. Geriatr. Med.* 29 753–772. 10.1016/j.cger.2013.07.003 24094295 PMC3821397

[B46] Ruthirakuhan M. Herrmann N. Vieira D. Gallagher D. Lanctôt K. L. (2019). The roles of apathy and depression in predicting Alzheimer disease: a longitudinal analysis in older adults with mild cognitive impairment. *Am. J. Geriatr. Psychiatry* 27 873–882. 10.1016/j.jagp.2019.02.003 30910421 PMC6646066

[B47] Santiago J. A. Potashkin J. A. (2021). The impact of disease comorbidities in Alzheimer’s disease. *Front. Aging Neurosci.* 13:631770. 10.3389/fnagi.2021.631770 33643025 PMC7906983

[B48] Siemers E. (2021). The Changing Diagnostic Criteria for AD, Including Early and Asymptomatic Disease Stages and their Impact on Clinical Trial Design. Available online at: https://www.ema.europa.eu/en/documents/presentation/presentation-changing-diagnostic-criteria-alzheimers-disease-including-early-asymptomatic-disease_en.pdf (accessed September 20, 2021).

[B49] Small G. (2017). Breaking down barriers to early Elzheimer’s disease diagnosis. *Today’s Geriatric Med.* 10:28.

[B50] Speechly C. M. Bridges-Webb C. Passmore E. (2008). The pathway to dementia diagnosis. *Med. J. Aust.* 189 487–489. 18976188 10.5694/j.1326-5377.2008.tb02140.x

[B51] Sperling R. Mormino E. Johnson K. (2014). The evolution of preclinical Alzheimer’s disease: implications for prevention trials. *Neuron* 84 608–622. 25442939 10.1016/j.neuron.2014.10.038 PMC4285623

[B52] Sperling R. A. Aisen P. S. Beckett L. A. Bennett D. A. Craft S. Fagan A. M. (2011). Toward defining the preclinical stages of Alzheimer’s disease: recommendations from the National Institute on Aging-Alzheimer’s Association workgroups on diagnostic guidelines for Alzheimer’s disease. *Alzheimers Dement* 7 280–292. 21514248 10.1016/j.jalz.2011.03.003 PMC3220946

[B53] Stark S. L. Roe C. M. Grant E. A. Hollingsworth H. Benzinger T. L. Fagan A. M. (2013). Preclinical Alzheimer disease and risk of falls. *Neurology* 81 437–443. 10.1212/wnl.0b013e31829d8599 23803314 PMC3776538

[B54] van Vliet D. de Vugt M. E. Bakker C. Pijnenburg Y. A. Vernooij-Dassen M. J. Koopmans R. T. (2013). Time to diagnosis in young-onset dementia as compared with late-onset dementia. *Psychol. Med.* 43 423–432. 10.1017/s0033291712001122 22640548

[B55] Vassilaki M. Aakre J. A. Cha R. H. Kremers W. K. St Sauver J. L. Mielke M. M. (2015). Multimorbidity and risk of mild cognitive impairment. *J. Am. Geriatr. Soc.* 63 1783–1790. 26311270 10.1111/jgs.13612 PMC4607039

[B56] Vassilaki M. Aakre J. A. Kremers W. K. Mielke M. M. Geda Y. E. Alhurani R. E. (2019). The association of multimorbidity with preclinical AD stages and SNAP in cognitively unimpaired persons. *J. Gerontol. A Biol. Sci. Med. Sci.* 74 877–883. 10.1093/gerona/gly149 30124772 PMC6521911

[B57] Velayudhan L. Poppe M. Archer N. Proitsi P. Brown R. G. Lovestone S. (2010). Risk of developing dementia in people with diabetes and mild cognitive impairment. *Br. J. Psychiatry* 196 36–40. 20044657 10.1192/bjp.bp.109.067942

[B58] Vicini Chilovi B. Conti M. Zanetti M. Mazzù I. Rozzini L. Padovani A. (2009). Differential impact of apathy and depression in the development of dementia in mild cognitive impairment patients. *Dement. Geriatr. Cogn. Disord* 27 390–398. 10.1159/000210045 19339777

[B59] Wackerbarth S. B. Johnson M. M. (2002). The carrot and the stick: benefits and barriers in getting a diagnosis. *Alzheimer Dis. Assoc. Disord.* 16 213–220. 10.1097/00002093-200210000-00002 12468895

[B60] Wang Y. Emre C. Gyllenhammar-Schill H. Fjellman K. Eyjolfsdottir H. Eriksdotter M. (2021). Cerebrospinal fluid inflammatory markers in Alzheimer’s disease: influence of comorbidities. *Curr. Alzheimer Res.* 18 157–170. 10.2174/1567205018666210330162207 33784960

[B61] Wasey J. O. Druker V. Lee E. (2021). *ICD: Comorbidity Calculations and Tools for ICD-9 and ICD-10 Codes.* Available online at: https://jackwasey.github.io/icd/index.html (accessed September 27, 2021).

[B62] Wilkinson D. Stave C. Keohane D. Vincenzino O. (2004). The role of general practitioners in the diagnosis and treatment of Alzheimer’s disease: a multinational survey. *J. Int. Med. Res.* 32 149–159. 10.1177/147323000403200207 15080018

[B63] World Health Organization [WHO] (2021). *Dementia Fact Sheet.* Geneva: World Health Organization.

[B64] Xu W. Caracciolo B. Wang H. X. Winblad B. Bäckman L. Qiu C. (2010). Accelerated progression from mild cognitive impairment to dementia in people with diabetes. *Diabetes Metab. Res. Rev.* 59 2928–2935. 10.2337/db10-0539 20713684 PMC2963552

[B65] Zhao M. Lv X. Tuerxun M. He J. Luo B. Chen W. (2016). Delayed help seeking behavior in dementia care: preliminary findings from the Clinical Pathway for Alzheimer’s Disease in China (CPAD) study. *Int. Psychogeriatr.* 28 211–219. 10.1017/s1041610215000940 26138923

