# Supplementary material for: Comorbidity Trajectories Associated With Alzheimer’s Disease: A Matched Case-Control Study in a United States Claims Database
Source: Front Neurosci. 2021 Oct 8;15:749305. doi: 10.3389/fnins.2021.749305 (PMC8531650; doi:10.3389/fnins.2021.749305)
Supplement: Supplementary file 1 [file Table_1.pdf]

Supplementary Material

Supplementary Table 1. Comorbidities with ≥5% prevalence among both cases and controls

3-digit ICD grouping	Description	Overall N=186,064		Cases N=93,032		Controls N=93,032		OR from GEE model (95% CI)*
		Number of patients	Prevalence	Number of patients	Prevalence	Number of patients	Prevalence	
**CLUSTER 1**								
401	Essential hypertension	138,662	74.5	77,804	83.6	60,858	65.4	2.08 (2.05, 2.10)
780	General symptoms	124,434	66.9	80,259	86.3	44,175	47.5	3.14 (3.10, 3.18)
786	Symptoms involving respiratory system and other chest symptoms	122,161	65.6	70,509	75.8	51,652	55.5	1.85 (1.82, 1.87)
719	Other and unspecified disorders of joint	99,270	53.3	59,076	63.5	40,194	43.2	1.85 (1.83, 1.88)
427	Cardiac dysrhythmias	71,654	38.5	41,652	44.8	30,002	32.2	1.61 (1.59, 1.64)
599	Other disorders of urethra and urinary tract	67,782	36.4	42,256	45.4	25,526	27.4	1.90 (1.87, 1.93)
959	Injury, other and unspecified	50,113	26.9	34,178	36.7	15,935	17.1	2.33 (2.39, 2.49)
276	Disorders of fluid, electrolyte, and acid-base balance	47,827	25.7	30,866	33.2	16,961	18.2	1.85 (1.82, 1.89)
781	Symptoms involving nervous and musculoskeletal systems	47,290	25.4	33,227	35.7	14,063	15.1	2.60 (2.54, 2.65)
110	Dermatophytosis	46,455	25.0	29,717	31.9	16,738	18.0	1.90 (1.86, 1.94)
428	Heart failure	41,215	22.2	23,627	25.4	17,588	18.9	1.42 (1.39, 1.45)
728	Disorders of muscle, ligament, and fascia	38,419	20.6	25,340	27.2	13,079	14.1	1.96 (1.91, 2.00)
799	Other ill-defined and unknown causes of morbidity and mortality	34,675	18.6	22,104	23.8	12,571	13.5	1.73 (1.69, 1.78)
294	Persistent mental disorders due to conditions classified elsewhere	31,899	17.1	29,356	31.6	2,543	2.7	8.55 (8.15, 8.96)
331	Other cerebral degenerations	29,056	15.6	24,132	25.9	4,924	5.3	4.96 (4.80, 5.13)
348	Other conditions of brain	20,061	10.8	15,996	17.2	4,065	4.4	3.71 (3.58, 3.86)
298	Other nonorganic psychoses	19,620	10.5	16,789	18.0	2,831	3.0	4.98 (4.77, 5.20)

3-digit ICD grouping	Description	Overall N=186,064		Cases N=93,032		Controls N=93,032		OR from GEE model (95% CI)*
		Number of patients	Prevalence	Number of patients	Prevalence	Number of patients	Prevalence	
293	Transient mental disorders due to conditions classified elsewhere	12,712	6.8	10,664	11.5	2,048	2.2	4.16 (3.95, 4.39)
**CLUSTER 2**								
486	Pneumonia, organism unspecified	32,497	17.5	19,137	20.6	13,360	14.4	1.42 (1.38, 1.45)
593	Other disorders of kidney and ureter	28,535	15.3	16,865	18.1	11,670	12.5	1.49 (1.45, 1.53)
585	Chronic kidney disease (CKD)	24,790	13.3	14,570	15.7	10,220	11.0	1.42 (1.38, 1.46)
511	Pleurisy	21,283	11.4	11,634	12.5	9,649	10.4	1.11 (1.08, 1.14)
584	Acute kidney failure	19,739	10.6	12,168	13.1	7,571	8.1	1.45 (1.41, 1.50)
995	Certain adverse effects not elsewhere classified	16,905	9.1	10,446	11.2	6,459	6.9	1.57 (1.52, 1.62)
491	Chronic bronchitis	16,678	9.0	9,342	10.0	7,336	7.9	1.35 (1.31, 1.40)
796	Other non-specific abnormal findings	15,535	8.4	9,601	10.3	5,934	6.4	1.54 (1.49, 1.59)
403	Hypertensive chronic kidney disease	13,385	7.2	8,188	8.8	5,197	5.6	1.49 (1.43, 1.55)
410	Acute myocardial infarction	11,661	6.3	6,764	7.3	4,897	5.3	1.36 (1.31, 1.41)
288	Diseases of white blood cells	11,246	6.0	7,007	7.5	4,239	4.6	1.50 (1.44, 1.56)
38	Septicemia	11,128	6.0	6,935	7.5	4,193	4.5	1.49 (1.43, 1.55)
514	Pulmonary congestion and hypostasis	11,117	6.0	6,596	7.1	4,521	4.9	1.35 (1.29, 1.40)
453	Other venous embolism and thrombosis	11,002	5.9	6,650	7.2	4,352	4.7	1.55 (1.48, 1.61)
V49	Other conditions influencing health status	10,535	5.7	6,469	7.0	4,066	4.4	1.51 (1.45, 1.58)
560	Intestinal obstruction without mention of hernia	9,899	5.3	6,101	6.6	3,798	4.1	1.62 (1.56, 1.70)
492	Emphysema	9,706	5.2	5,557	6.0	4,149	4.5	1.30 (1.25, 1.36)
41	Bacterial infection in conditions classified elsewhere and of unspecified site	9,619	5.2	6,379	6.9	3,240	3.5	1.81 (1.73, 1.90)
805	Fracture of vertebral column without mention of spinal cord injury	9,344	5.0	6,157	6.6	3,187	3.4	1.91 (1.82, 2.00)
**CLUSTER 3**								
784	Symptoms involving head and neck	53,436	28.7	35,459	38.11	17,977	19.33	2.25 (2.20, 2.29)
V57	Care involving use of rehabilitation procedures	31,398	16.9	20,260	21.8	11,138	11.0	1.89 (1.84, 1.93)
437	Other and ill-defined cerebrovascular disease	25,825	13.9	19,588	21.1	6,237	6.7	3.12 (3.02, 3.21)

3-digit ICD grouping	Description	Overall N=186,064		Cases N=93,032		Controls N=93,032		OR from GEE model (95% CI)*
		Number of patients	Prevalence	Number of patients	Prevalence	Number of patients	Prevalence	
300	Anxiety, dissociative and somatoform disorders	25,601	13.8	18,388	19.8	7,213	7.8	2.60 (2.53, 2.68)
783	Symptoms concerning nutrition, metabolism, and development	25,469	13.7	17,988	19.3	7,481	8.0	2.46 (2.40, 2.54)
311	Depressive disorder, not elsewhere classified	24,954	13.4	18,995	20.4	5,959	6.4	3.18 (3.08, 3.28)
435	Transient cerebral ischemia	23,894	12.8	16,719	18.0	7,175	7.7	2.46 (2.39, 2.53)
V15	Other personal history presenting hazards to health	23,819	12.8	16,218	17.4	7,601	8.2	2.03 (1.97, 2.09)
434	Occlusion of cerebral arteries	22,024	11.8	15,560	16.7	6,464	7.0	2.50 (2.42, 2.58)
924	Contusion of lower limb and of other and unspecified sites	20,998	11.3	14,146	15.2	6,852	7.4	2.12 (2.06, 2.19)
707	Chronic ulcer of skin	20,073	10.8	12,617	13.6	7,456	8.0	1.73 (1.67, 1.78)
458	Hypotension	19,292	10.3	12,763	13.7	6,529	7.0	1.94 (1.88, 2.01)
436	Acute, but ill-defined, cerebrovascular disease	18,748	10.1	13,097	14.1	5,651	6.1	2.48 (2.39, 2.56)
296	Episodic mood disorders	14,363	7.7	11,130	12.0	3,233	3.5	3.61 (3.46, 3.76)
438	Late effects of cerebrovascular disease	13,705	7.4	9,890	10.6	3,815	4.1	2.53 (2.43, 2.64)
873	Other open wound of head	13,686	7.34	9,568	10.3	4,118	4.4	2.33 (2.25, 2.42)
V71	Observation and evaluation for suspected conditions not found	13,394	7.2	8,929	9.6	4,465	4.8	1.91 (1.84, 1.98)
920	Contusion of face, scalp, and neck except eye(s)	13,316	7.2	9,678	10.4	3,638	3.9	2.61 (2.51, 2.71)
266	Deficiency of B-complex components	10,870	5.8	7,724	8.3	3,146	3.4	2.29 (2.19, 2.39)
820	Fracture of neck of femur	10,777	5.8	7,349	7.9	3,428	3.7	2.07 (1.98, 2.16)
922	Contusion of trunk	10,421	5.6	7,277	7.8	3,144	3.4	2.38 (2.28, 2.48)
923	Contusion of upper limb	10,164	5.5	6,967	7.4	3,197	3.4	2.18 (2.09, 2.27)
**CLUSTER 4**								
702	Other dermatoses	59,353	31.9	31,337	33.7	28,016	30.1	1.14 (1.12, 1.16)
V72	Special investigations and examinations	58,328	31.3	32,509	34.9	25,819	27.8	1.30 (1.28, 1.32)
V43	Organ or tissue replaced by other means	46,529	25.0	26,430	28.4	20,099	21.6	1.36 (1.33, 1.39)
365	Glaucoma	37,941	20.4	21,110	22.7	16,831	18.1	1.38 (1.35, 1.41)

3-digit ICD grouping	Description	Overall N=186,064		Cases N=93,032		Controls N=93,032		OR from GEE model (95% CI)*
		Number of patients	Prevalence	Number of patients	Prevalence	Number of patients	Prevalence	
726	Peripheral enthesopathies and allied syndromes	37,928	20.4	21,434	23.0	16,494	17.7	1.36 (1.33, 1.39)
466	Acute bronchitis and bronchiolitis	36,542	19.6	20,648	22.2	15,894	17.1	1.36 (1.33, 1.39)
238	Neoplasm of uncertain behavior of other and unspecified sites and tissues	35,273	19.0	19,024	20.4	16,249	17.5	1.18 (1.15, 1.21)
692	Contact dermatitis and other eczema	33,947	18.2	19,668	21.1	14,279	15.4	1.42 (1.39, 1.45)
173	Other and unspecified malignant neoplasm of skin	31,753	17.1	17,211	18.5	14,542	15.6	1.24 (1.21, 1.27)
V10	Personal history of malignant neoplasm	31,692	17.0	17,229	18.5	14,463	15.5	1.22 (1.19, 1.25)
211	Benign neoplasm of other parts of digestive system	31,187	16.8	17,242	18.5	13,945	15.0	1.27 (1.24, 1.30)
V70	General medical examination	30,935	16.6	17,491	18.8	13,444	14.5	1.27 (1.24, 1.30)
465	Acute upper respiratory infections of multiple or unspecified sites	27,935	15.0	16,238	17.5	11,697	12.6	1.43 (1.39, 1.46)
379	Other disorders of eye	26,993	14.5	15,381	16.5	11,612	12.5	1.33 (1.29, 1.36)
216	Benign neoplasm of skin	24,930	13.4	13,323	14.3	11,607	12.5	1.12 (1.09, 1.15)
709	Other disorders of skin and subcutaneous tissue	24,367	13.1	13,550	14.6	10,817	11.6	1.26 (1.23, 1.30)
569	Other disorders of intestine	23,086	12.4	13,526	14.5	9,560	10.3	1.47 (1.43, 1.51)
461	Acute sinusitis	22,396	12.0	12,316	13.2	10,080	10.8	1.24 (1.21, 1.27)
477	Allergic rhinitis	22,378	12.0	13,007	14.0	9,371	10.1	1.39 (1.35, 1.43)
727	Other disorders of synovium, tendon, and bursa	20,856	11.2	11,474	12.3	9,382	10.1	1.23 (1.19, 1.26)
V67	Follow-up examination	20,597	11.1	11,598	12.5	8,999	9.7	1.28 (1.24, 1.32)
375	Disorders of lacrimal system	20,352	10.9	11,610	12.5	8,742	9.4	1.72 (1.67, 1.79)
706	Diseases of sebaceous glands	20,183	10.9	11,643	12.5	8,540	9.2	1.40 (1.36, 1.44)
553	Other hernia of abdominal cavity without mention of obstruction or gangrene	19,813	10.7	11,598	12.5	8,215	8.8	1.46 (1.42, 1.50)
372	Disorders of conjunctiva	19,805	10.6	11,704	12.6	8,101	8.7	1.45 (1.41, 1.49)
426	Conduction disorders	19,472	10.5	11,768	12.7	7,704	8.3	1.54 (1.49, 1.59)
280	Iron deficiency anemias	19,113	10.3	11,425	12.3	7,688	8.3	1.52 (1.47, 1.57)
402	Hypertensive heart disease	18,707	10.1	11,228	12.1	7,479	8.0	1.55 (1.50, 1.60)
493	Asthma	18,134	9.7	10,028	10.8	8,106	8.7	1.31 (1.27, 1.35)

3-digit ICD grouping	Description	Overall N=186,064		Cases N=93,032		Controls N=93,032		OR from GEE model (95% CI)*
		Number of patients	Prevalence	Number of patients	Prevalence	Number of patients	Prevalence	
459	Other disorders of circulatory system	16,873	9.1	10,362	11.1	6,511	7.0	1.59 (1.53, 1.64)
455	Hemorrhoids	16,547	8.9	9,780	10.5	6,767	7.3	1.47 (1.43, 1.52)
611	Other disorders of breast	16,394	8.8	9,203	9.9	7,191	7.7	1.31 (1.27, 1.35)
413	Angina pectoris	16,301	8.8	9,575	10.3	6,726	7.2	1.54 (1.48, 1.59)
739	Nonallopathic lesions, not elsewhere classified	15,525	8.3	8,066	8.7	7,459	8.0	1.06 (1.02, 1.09)
490	Bronchitis, not specified as acute or chronic	15,279	8.2	8,890	9.6	6,389	6.9	1.06 (1.02, 1.09)
373	Inflammation of eyelids	14,909	8.0	8,572	9.2	6,337	6.8	1.38 (1.34, 1.43)
558	Other and unspecified noninfectious gastroenteritis and colitis	14,429	7.8	8,919	9.6	5,510	5.9	1.66 (1.61, 1.72)
V03	Need for prophylactic vaccination and inoculation against bacterial diseases	14,400	7.7	8,695	9.3	5,705	6.1	1.51 (1.46, 1.56)
681	Cellulitis and abscess of finger and toe	14,065	7.6	8,708	9.4	5,357	5.8	1.65 (1.59, 1.71)
996	Complications peculiar to certain specified procedures	14,001	7.5	8,217	8.8	5,784	6.2	1.47 (1.42, 1.52)
239	Neoplasms of unspecified nature	13,958	7.5	7,688	8.3	6,270	6.7	1.25 (1.21, 1.30)
374	Other disorders of eyelids	13,810	7.4	7,770	8.4	6,040	6.5	1.32 (1.27, 1.37)
388	Other disorders of ear	13,784	7.4	8,203	8.8	5,581	6.0	1.50 (1.45, 1.55)
367	Disorders of refraction and accommodation	13,677	7.4	7,654	8.2	6,023	6.5	1.24 (1.20, 1.29)
473	Chronic sinusitis	13,621	7.3	8,058	8.7	5,563	6.0	1.44 (1.39, 1.49)
701	Other hypertrophic and atrophic conditions of skin	13,467	7.2	7,625	8.2	5,842	6.3	1.32 (1.28, 1.37)
411	Other acute and subacute forms of ischemic heart disease	12,856	6.9	7,670	8.2	5,186	5.6	1.58 (1.52, 1.65)
268	Vitamin D deficiency	12,568	6.8	8,045	8.7	4,523	4.9	1.70 (1.63, 1.77)
425	Cardiomyopathy	11,899	6.4	6,784	7.3	5,115	5.5	1.37 (1.32, 1.42)
847	Sprains and strains of other and unspecified parts of back	11,882	6.4	7,495	8.05	4,387	4.71	1.72 (1.65, 1.78)
574	Cholelithiasis	11,582	6.2	6,707	7.21	4,875	5.24	1.41 (1.35, 1.46)
386	Vertiginous syndromes and other disorders of vestibular system	11,479	6.2	7,125	7.66	4,354	4.68	1.70 (1.64, 1.77)

3-digit ICD grouping	Description	Overall N=186,064		Cases N=93,032		Controls N=93,032		OR from GEE model (95% CI)*
		Number of patients	Prevalence	Number of patients	Prevalence	Number of patients	Prevalence	
V06	Need for prophylactic vaccination and inoculation against combinations of diseases	11,355	6.1	6,738	7.24	4,617	4.96	1.47 (1.41, 1.52)
185	Malignant neoplasm of prostate	11,265	6.1	6,229	6.7	5,036	5.4	1.37 (1.32, 1.43)
595	Cystitis	10,927	5.9	6,850	7.4	4,077	4.4	1.65 (1.5, 1.72)
327	Organic sleep disorders	10,804	5.8	6,671	7.2	4,133	4.4	1.65 (1.58, 1.71)
462	Acute pharyngitis	10,634	5.7	5,952	6.4	4,682	5.0	1.31 (1.26, 1.37)
274	Gout	10,227	5.5	5,664	6.1	4,563	4.9	1.25 (1.20, 1.30)
441	Aortic aneurysm and dissection	10,201	5.5	5,802	6.2	4,399	4.7	1.40 (13.4, 1.46)
355	Mononeuritis of lower limb	10,045	5.4	6,199	6.7	3,846	4.1	1.62 (1.55, 1.69)
840	Sprains and strains of shoulder and upper arm	9,878	5.3	5,805	6.2	4,073	4.4	1.47 (1.41, 1.53)
412	Old myocardial infarction	9,863	5.3	6,013	6.5	3,850	4.1	1.58 (1.51, 1.65)
625	Pain and other symptoms associated with female genital organs	9,690	5.2	5,806	6.2	3,884	4.2	1.51 (1.45, 1.57)
53	Herpes zoster	9,372	5.0	5,403	5.8	3,969	4.3	1.41 (1.35, 1.47)
592	Calculus of kidney and ureter	9,331	5.0	5,559	6.0	3,772	4.1	1.49 (1.43, 1.56)
**CLUSTER 5**								
272	Disorders of lipoid metabolism	101,911	54.8	57,906	62.2	44,005	47.3	1.60 (1.58, 1.62)
729	Other disorders of soft tissues	83,444	44.8	49,899	53.6	33,545	36.1	1.76 (1.74, 1.79)
366	Cataract	82,036	44.1	45,372	48.8	36,664	39.4	1.32 (1.3, 1.34)
715	Osteoarthrosis and allied disorders	80,022	43.0	47,016	50.5	33,006	35.5	1.66 (1.64, 1.68)
724	Other and unspecified disorders of back	71,981	38.7	42,506	45.7	29,475	31.7	1.67 (1.65,1.70)
789	Other symptoms involving abdomen and pelvis	70,320	37.8	41,863	44.0	28,457	30.6	1.73 (1.70, 1.75)
414	Other forms of chronic ischemic heart disease	66,130	35.5	37,906	40.7	28,224	30.3	1.64 (1.61, 1.66)
787	Symptoms involving digestive system	64,251	34.5	39,632	42.6	24,619	26.5	1.84 (1.81, 1.88)
V58	Encounter for other and unspecified procedures and aftercare	63,772	34.3	37,213	34.0	26,559	28.5	1.53 (1.51, 1.56)
733	Other disorders of bone and cartilage	60,220	32.4	35,141	37.8	25,079	27.0	1.57 (1.54, 1.60)
782	Symptoms involving skin and other integumentary tissue	58,952	31.7	35,630	38.3	23,322	25.1	1.66 (1.64, 1.69)

3-digit ICD grouping	Description	Overall N=186,064		Cases N=93,032		Controls N=93,032		OR from GEE model (95% CI)*
		Number of patients	Prevalence	Number of patients	Prevalence	Number of patients	Prevalence	
250	Diabetes mellitus	53,171	28.6	30,073	32.3	23,098	24.8	1.68 (1.65, 1.71)
788	Symptoms involving urinary system	52,742	28.3	33,397	35.9	19,345	20.8	1.94 (1.90, 1.97)
V04	Need for prophylactic vaccination and inoculation against certain diseases	51,621	27.7	29,855	32.1	21,766	23.4	1.44 (1.41, 1.46)
362	Other retinal disorders	49,805	26.8	28,387	30.5	21,418	23.0	1.43 (1.41, 1.46)
785	Symptoms involving cardiovascular system	49,111	26.4	28,334	30.5	20,777	22.3	1.44 (1.41, 1.47)
530	Diseases of esophagus	49,013	26.3	28,788	30.9	20,225	21.7	1.56 (1.53, 1.59)
793	Non-specific (abnormal) findings on radiological and other examination of body structure	48,479	26.1	28,358	30.5	20,121	21.6	1.45 (1.42, 1.48)
285	Other and unspecified anemias	48,234	25.9	29,199	31.4	19,035	20.5	1.63 (1.60, 1.66)
518	Other diseases of lung	46,216	24.8	26,596	28.6	19,620	21.1	1.41 (1.38, 1.44)
424	Other diseases of endocardium	44,749	24.1	26,125	28.1	18,624	20.0	1.45 (1.43, 1.48)
722	Intervertebral disc disorders	44,055	23.7	26,276	28.2	17,779	19.1	1.60 (1.57, 1.63)
794	Non-specific abnormal results of function studies	42,948	23.1	25,955	27.9	16,993	18.3	1.61 (1.58, 1.65)
790	Non-specific findings on examination of blood	41,454	22.3	24,661	26.5	16,793	18.1	1.49 (1.46, 1.52)
433	Occlusion and stenosis of precerebral arteries	40,440	21.7	25,614	27.5	14,826	15.9	1.79 (1.75, 1.83)
562	Diverticula of intestine	38,712	20.8	22,321	24.0	16,391	17.6	1.44 (1.41, 1.47)
244	Acquired hypothyroidism	38,087	20.5	22,754	24.5	15,333	16.5	1.62 (1.59, 1.66)
389	Hearing loss	37,716	203	22,885	24.6	14,831	15.9	1.67 (1.64, 1.71)
496	Chronic airway obstruction, not elsewhere classified	37,651	20.2	21,720	23.3	15,931	17.1	1.47 (1.44, 1.50)
429	Ill-defined descriptions and complications of heart disease	36,462	19.6	22,149	23.8	14,313	15.4	1.62 (1.58, 1.66)
V45	Other postprocedural states	35,833	19.3	20,928	22.5	14,905	16.0	1.52 (1.49, 1.56)
564	Functional digestive disorders, not elsewhere classified	33,810	18.2	21,843	23.5	11,967	12.9	1.99 (1.95, 2.04)
721	Spondylosis and allied disorders	33,716	18.1	20,503	22.0	13,213	14.2	1.66 (1.62, 1.69)
380	Disorders of external ear	32,857	17.7	19,330	20.8	13,527	14.5	1.49 (1.45, 1.52)

3-digit ICD grouping	Description	Overall N=186,064		Cases N=93,032		Controls N=93,032		OR from GEE model (95% CI)*
		Number of patients	Prevalence	Number of patients	Prevalence	Number of patients	Prevalence	
440	Atherosclerosis	30,406	16.3	18,564	120.0	11,842	12.7	1.58 (1.54, 1.62)
682	Other cellulitis and abscess	29,291	15.7	18,002	19.4	11,289	12.1	1.70 (1.66, 1.74)
443	Other peripheral vascular disease	29,215	15.7	17,991	19.3	11,224	12.1	1.63 (1.59, 1.67)
723	Other disorders of cervical region	29,183	15.7	17,982	19.3	11,201	12.0	1.68 (1.64, 1.72)
600	Hyperplasia of prostate	29,144	15.7	17,336	18.6	11,808	12.7	1.60 (1.56, 1.64)
V12	Personal history of certain other diseases	28,792	15.5	17,666	19.0	11,126	12.0	1.70 (1.66, 1.74)
716	Other and unspecified arthropathies	28,064	15.1	17,131	18.4	10,933	11.8	1.67 (1.63, 1.71)
703	Diseases of nail	26,795	14.4	17,132	18.4	9,663	10.4	1.82 (1.78, 1.87)
535	Gastritis and duodenitis	23,421	12.6	14,279	15.4	9,142	9.8	1.69 (1.64, 1.73)
578	Gastrointestinal hemorrhage	21,254	11.4	12,872	13.8	8,382	9.0	1.59 (1.55, 1.64)
V54	Other orthopedic aftercare	18,764	10.1	11,788	12.7	6,976	7.5	1.68 (1.63, 1.73)
735	Acquired deformities of toe	16,404	8.8	10,301	11.1	6,103	6.6	1.73 (1.67, 1.79)
338	Pain, not elsewhere classified	14,834	8.0	9,557	10.3	5,277	5.7	1.82 (1.75, 1.89)
596	Other disorders of bladder	14,444	7.8	9,243	9.9	5,201	5.6	1.88 (1.81, 1.95)
368	Visual disturbances	14,121	7.6	8,878	9.5	5,243	5.6	1.73 (1.67, 1.79)
356	Hereditary and idiopathic peripheral neuropathy	13,455	7.2	8,604	9.3	4,851	5.2	1.84 (1.77, 1.91)
281	Other deficiency anemias	12,172	6.5	8,002	8.6	4,170	4.5	1.98 (1.91, 2.07)

AD, Alzheimer's disease; CI, confidence interval; GEE, generalized estimating equation; ICD, International Classification of Disease, OR, odds ratio

*ORs comparing AD versus controls at the mean follow-up time prior to AD diagnosis

Supplementary Figure 1. Comorbidities with highest odds ratios in AD cases compared to controls in the period prior to index date

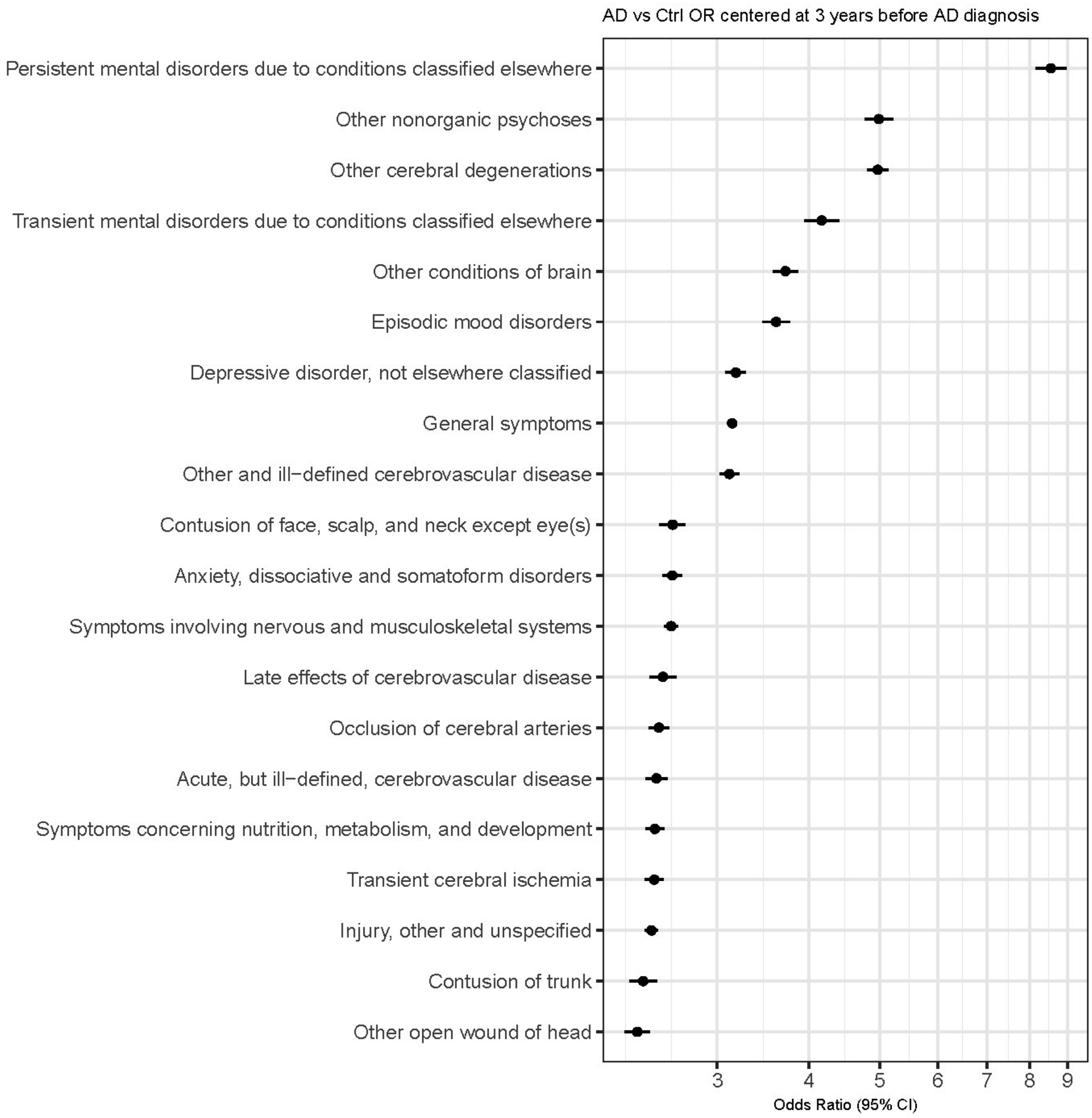

AD, Alzheimer's disease; ctrl, control; OR, odds ratio

Supplementary Figure 2. Distribution of comorbidities in a) the first and second principal components and b) the first and third principal components

a) First and second principal components

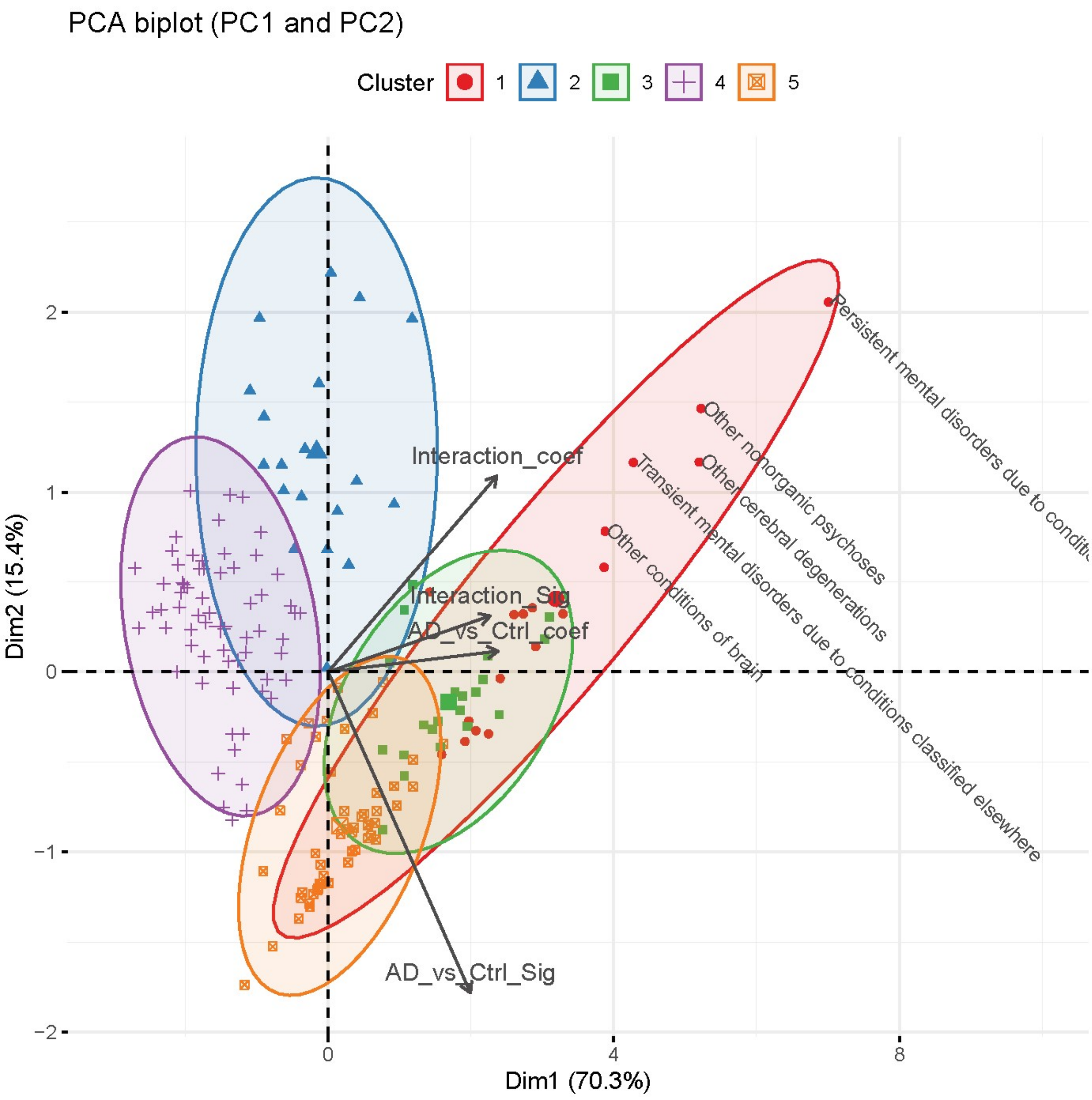

b) First and third principal components

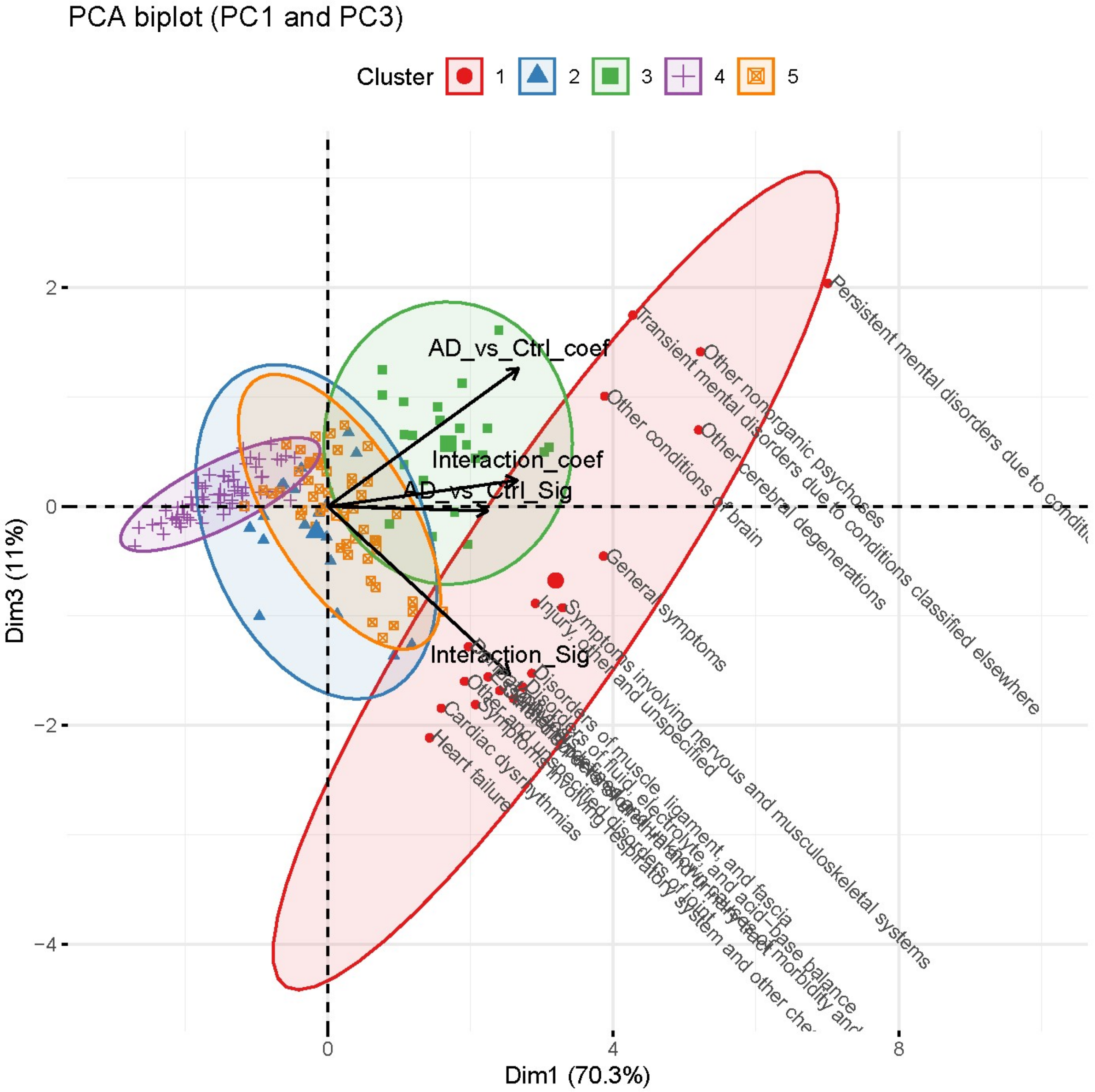

AD, Alzheimer's disease; ctrl, control; PC, principal component; PCA, principal component analysis

Dim1, Dim2, Dim3 = principal components 1,2 3 respectively. Numbers in brackets are proportion or variability explained by each principal component.

AD_versus_ctrol_Coef = size of overall comorbidity prevalence difference between cases and controls.
AD_versus_ctrol_Sig = p-value for overall comorbidity prevalence difference between cases and controls.
Interaction_Coef = size of comorbidity trajectory over time difference between cases and controls.
Interaction_Sig = p-value for comorbidity trajectory over time difference between cases and controls.

Supplementary Figure 3. Main clusters of comorbidities from the hierarchical cluster analysis conducted on the four principal components

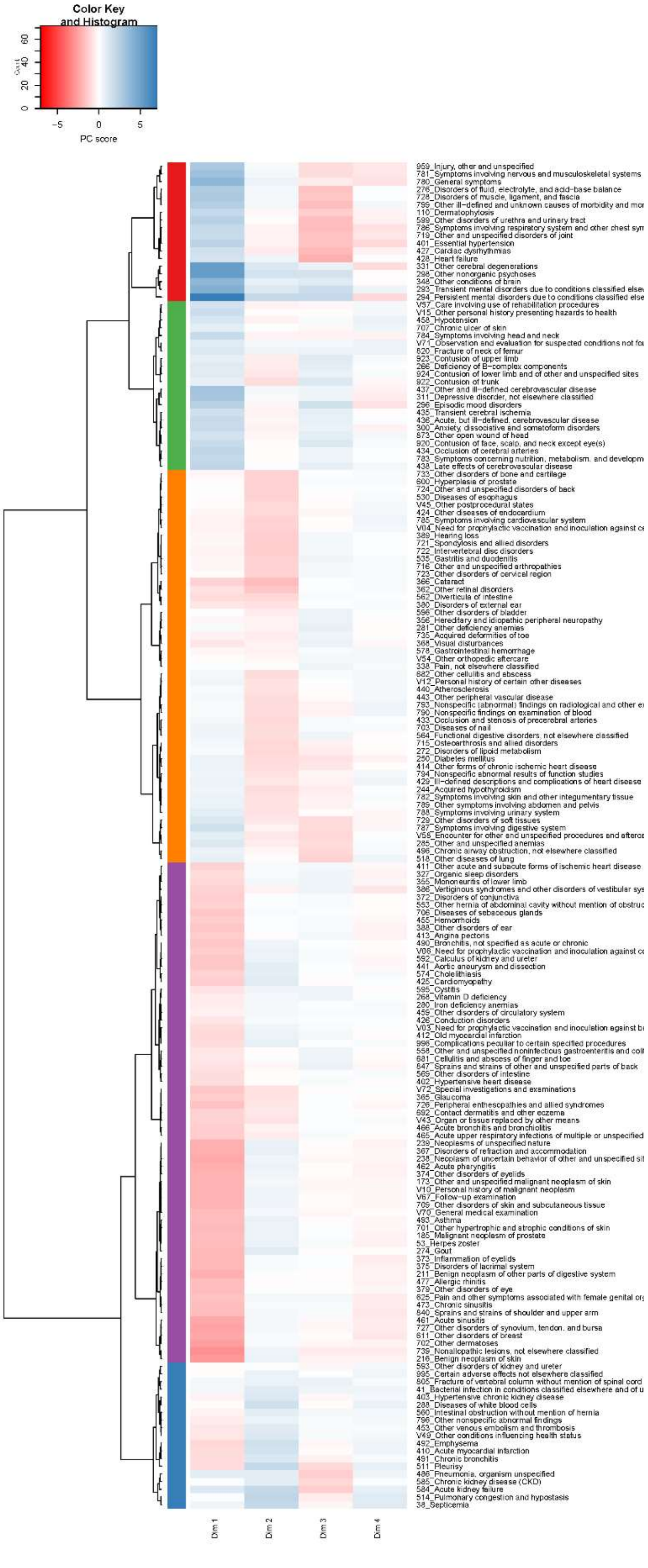

Cluster 1 (red), Cluster 2 (green), Cluster 3 (orange), cluster 4 (purple), Cluster 5 (blue).

Supplementary Figure 4. Time trajectories of comorbidities during the 5-year period before AD diagnosis between AD cases and controls in different clusters

a) Cluster 1

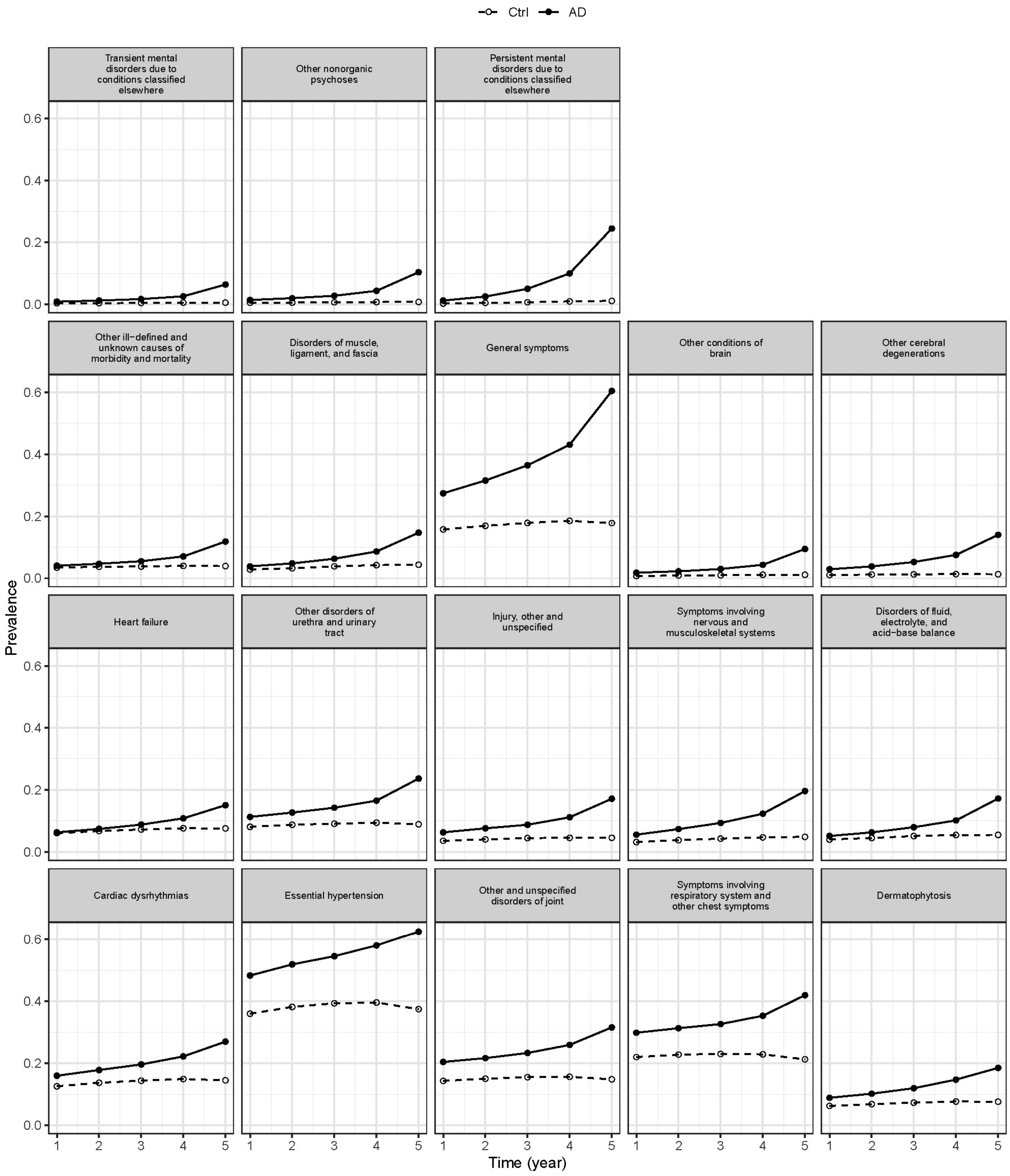

b) Cluster 2

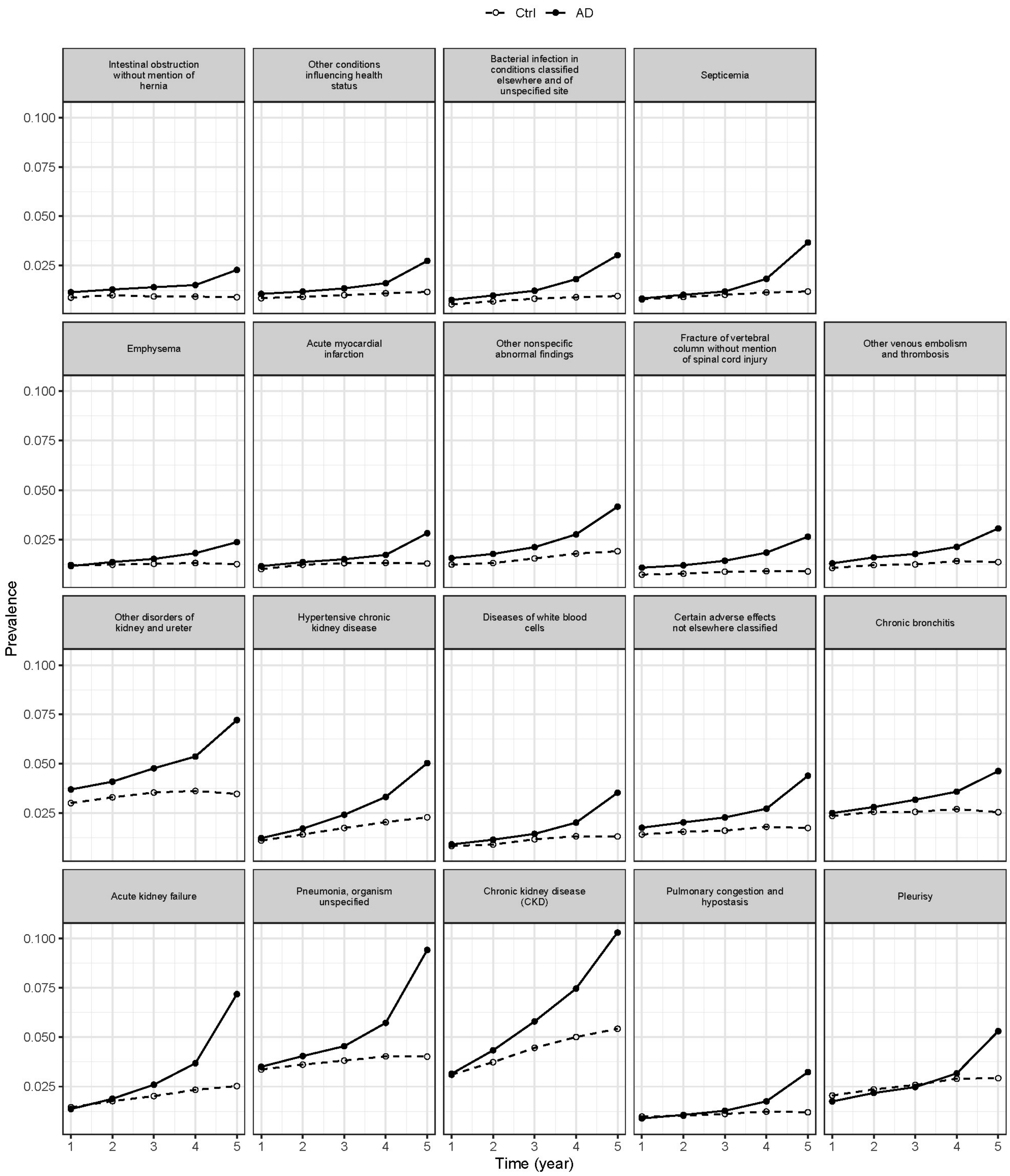
Ctrl
AD
Intestinal obstruction without mention of hernia
Other conditions influencing health status
Bacterial infection in conditions classified elsewhere and of unspecified site
Septicemia
Emphysema
Acute myocardial infarction
Other nonspecific abnormal findings
Fracture of vertebral column without mention of spinal cord injury
Other venous embolism and thrombosis
Other disorders of kidney and ureter
Hypertensive chronic kidney disease
Diseases of white blood cells
Certain adverse effects not elsewhere classified
Chronic bronchitis
Acute kidney failure
Pneumonia, organism unspecified
Chronic kidney disease (CKD)
Pulmonary congestion and hypostasis
Pleurisy
Prevalence
0.100
0.075
0.050
0.025
1
2
3
4
5
Time (year)

c) Cluster 3

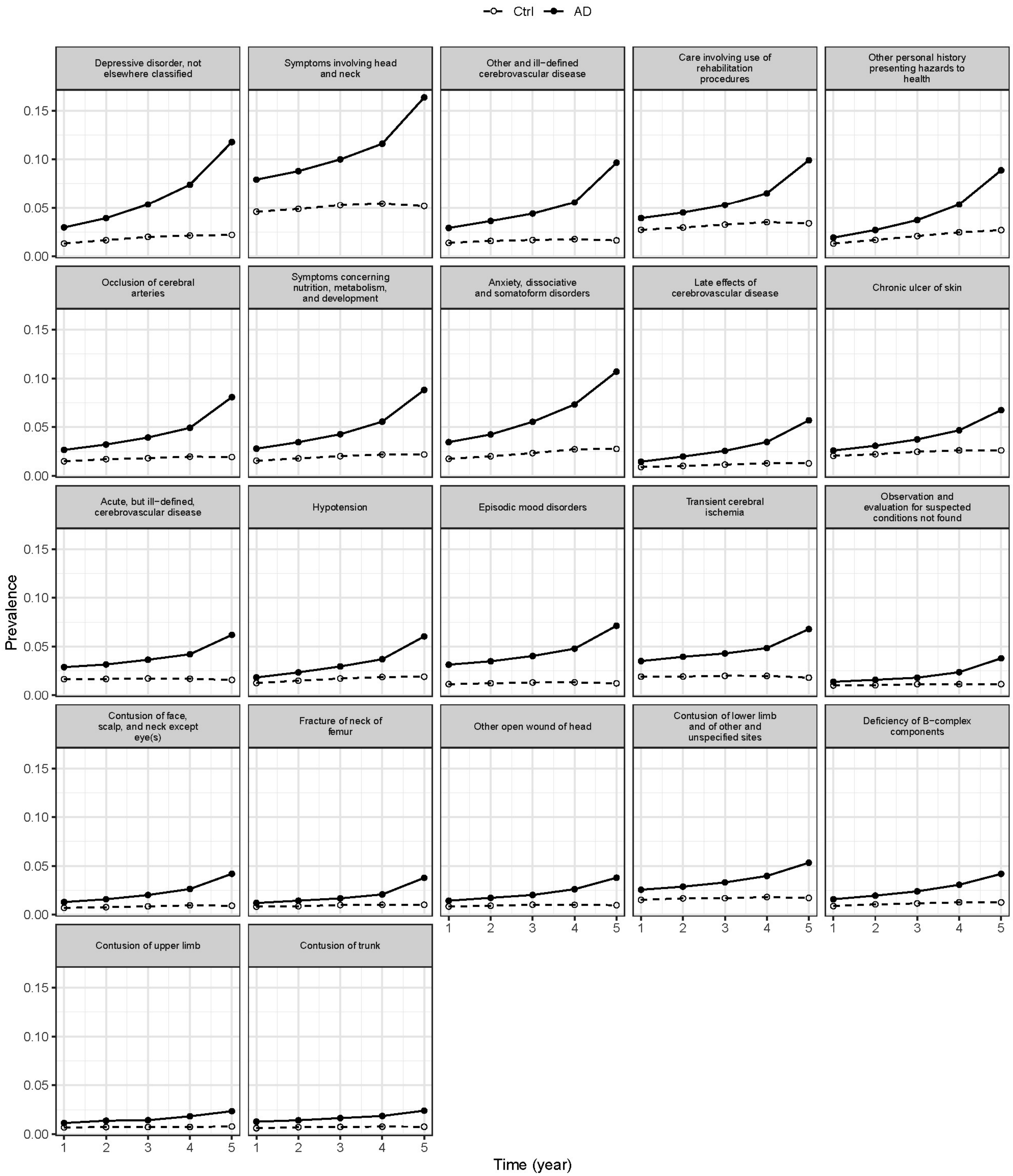
Ctrl
AD
Depressive disorder, not elsewhere classified
Symptoms involving head and neck
Other and ill−defined cerebrovascular disease
Care involving use of rehabilitation procedures
Other personal history presenting hazards to health
Occlusion of cerebral arteries
Symptoms concerning nutrition, metabolism, and development
Anxiety, dissociative and somatoform disorders
Late effects of cerebrovascular disease
Chronic ulcer of skin
Acute, but ill−defined, cerebrovascular disease
Hypotension
Episodic mood disorders
Transient cerebral ischemia
Observation and evaluation for suspected conditions not found
Contusion of face, scalp, and neck except eye(s)
Fracture of neck of femur
Other open wound of head
Contusion of lower limb and of other and unspecified sites
Deficiency of B−complex components
Contusion of upper limb
Contusion of trunk
Prevalence
0.00
0.05
0.10
0.15
1
2
3
4
5
Time (year)

d) Cluster 4

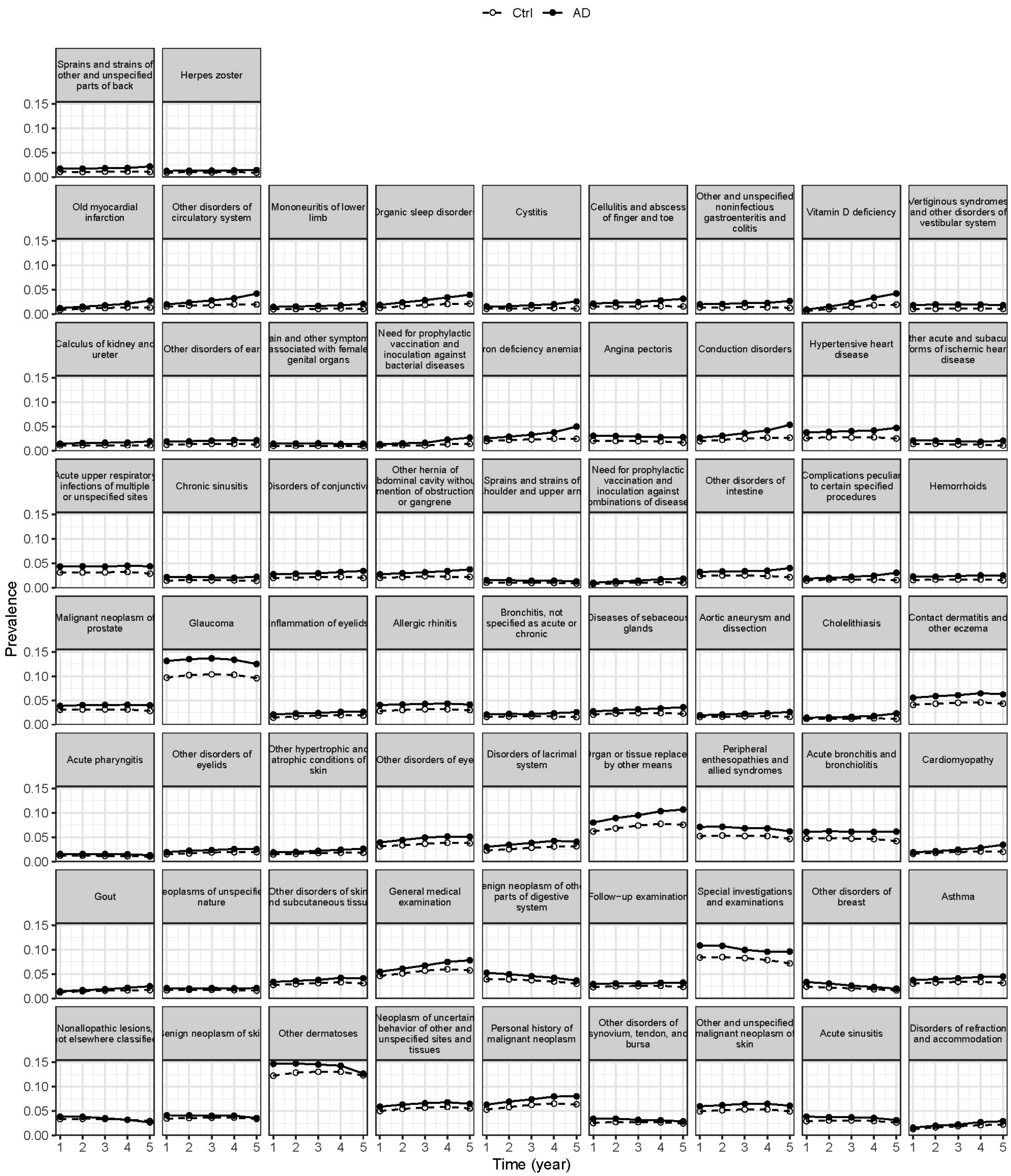
Ctrl
AD
Sprains and strains of other and unspecified parts of back
Herpes zoster
Old myocardial infarction
Other disorders of circulatory system
Mononeuritis of lower limb
Organic sleep disorders
Cystitis
Cellulitis and abscess of finger and toe
Other and unspecified noninfectious gastroenteritis and colitis
Vitamin D deficiency
Vertiginous syndromes and other disorders of vestibular system
Calculus of kidney and ureter
Other disorders of ear
Pain and other symptoms associated with female genital organs
Need for prophylactic vaccination and inoculation against bacterial diseases
Iron deficiency anemias
Angina pectoris
Conduction disorders
Hypertensive heart disease
Other acute and subacute forms of ischemic heart disease
Acute upper respiratory infections of multiple or unspecified sites
Chronic sinusitis
Disorders of conjunctiva
Other hernia of abdominal cavity without mention of obstruction or gangrene
Sprains and strains of shoulder and upper arm
Need for prophylactic vaccination and inoculation against combinations of diseases
Other disorders of intestine
Complications peculiar to certain specified procedures
Hemorrhoids
Malignant neoplasm of prostate
Glaucoma
Inflammation of eyelids
Allergic rhinitis
Bronchitis, not specified as acute or chronic
Diseases of sebaceous glands
Aortic aneurysm and dissection
Cholelithiasis
Contact dermatitis and other eczema
Acute pharyngitis
Other disorders of eyelids
Other hypertrophic and atrophic conditions of skin
Other disorders of eye
Disorders of lacrimal system
Organ or tissue replaced by other means
Peripheral enthesopathies and allied syndromes
Acute bronchitis and bronchiolitis
Cardiomyopathy
Gout
Neoplasms of unspecified nature
Other disorders of skin and subcutaneous tissue
General medical examination
Benign neoplasm of other parts of digestive system
Follow−up examination
Special investigations and examinations
Other disorders of breast
Asthma
Nonallopathic lesions, not elsewhere classified
Benign neoplasm of skin
Other dermatoses
Neoplasm of uncertain behavior of other and unspecified sites and tissues
Personal history of malignant neoplasm
Other disorders of synovium, tendon, and bursa
Other and unspecified malignant neoplasm of skin
Acute sinusitis
Disorders of refraction and accommodation
0.15
0.10
0.05
0.00
Prevalence
1 2 3 4 5
Time (year)

e) Cluster 5

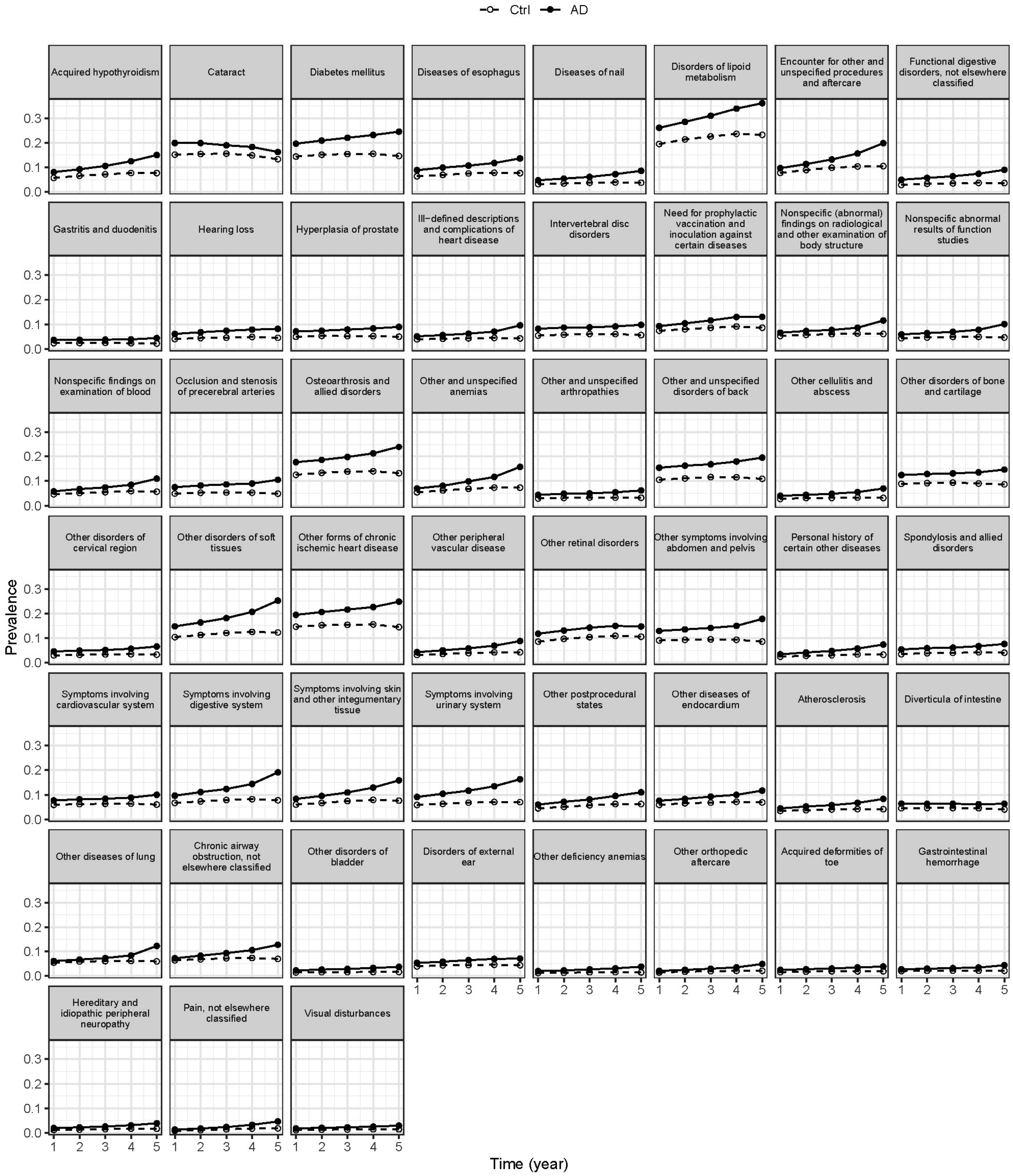

AD, Alzheimer’s disease; ctrl, control
